# Climate change, air pollution and maternal and newborn health: An overview of reviews of health outcomes

**DOI:** 10.7189/jogh.14.04128

**Published:** 2024-05-24

**Authors:** Francesca Conway, Anayda Portela, Veronique Filippi, Doris Chou, Sari Kovats

**Affiliations:** 1 World Health Organization, Department of Maternal, Newborn, Child and Adolescent Health and Ageing, Geneva, Switzerland; 2 London School of Hygiene and Tropical Medicine, Faculty of Epidemiology and Population Health, London, United Kingdom; 3 UNDP/UNFPA/UNICEF/WHO/The World Bank Special Programme of Research, Development and Research Training in Human Reproduction (HRP), World Health Organization, Department of Sexual and Reproductive Health, Geneva, Switzerland; 4 London School of Hygiene and Tropical Medicine, NIHR Health Protection Research Unit in Environmental Change and Health, London, United Kingdom

## Abstract

**Background:**

Climate change represents a fundamental threat to human health, with pregnant women and newborns being more susceptible than other populations. In this review, we aimed to describe the current landscape of available epidemiological evidence on key climate risks on maternal and newborn health (MNH).

**Methods:**

We sought to identify published systematic and scoping reviews investigating the impact of different climate hazards and air pollution on MNH outcomes. With this in mind, we developed a systematic search strategy based on the concepts of ‘climate/air pollution hazards, ‘maternal health,’ and ‘newborn health,’ with restrictions to reviews published between 1 January 2010 and 6 February 2023, but without geographical or language restriction. Following full text screening and data extraction, we synthesised the results using narrative synthesis.

**Results:**

We found 79 reviews investigating the effects of climate hazards on MNH, mainly focussing on outdoor air pollution (n = 47, 59%), heat (n = 24, 30%), and flood/storm disasters (n = 7, 9%). Most were published after 2015 (n = 60, 76%). These reviews had consistent findings regarding the positive association of exposure to heat and to air pollution with adverse birth outcomes, particularly preterm birth. We found limited evidence for impacts of climate-related food and water security on MNH and did not identify any reviews on climate-sensitive infectious diseases and MNH.

**Conclusions:**

Climate change could undermine recent improvements in maternal and newborn health. Our review provides an overview of key climate risks to MNH. It could therefore be useful to the MNH community to better understand the MNH needs for each climate hazard and to strengthen discussions on evidence and research gaps and potential actions. Despite the lack of comprehensive evidence for some climate hazards and for many maternal, perinatal, and newborn outcomes, we observed repeated findings of the impact of heat and air pollutants on birth outcomes, particularly preterm birth. It is time for policy dialogue to follow to specifically design climate policy and actions to protect the needs of MNH.

Climate change represents a fundamental threat to human health, increasing the vulnerability of populations to the coexisting geopolitical, energy, and cost-of-living crises [1,2]. According to the World Health Organization (WHO), between 2030 and 2050, climate change is projected to cause approximately 250 000 additional deaths per year from malnutrition, malaria, diarrhoea, and heat stress alone [2]. In fact, it is already impacting health in several ways, through the increase in extreme weather; the disruption of food systems; increases in zoonoses and food-, water- and vector-borne diseases; and by undermining many of the social determinants for health, such as livelihoods, equality, and access to health care and social support structures.

While all people are exposed to climate change, some groups are more affected or are particularly susceptible to negative health impacts. For example, pregnant and postpartum women, infants, and children have been found to have heightened vulnerability to climate risks due to a set of physiological, clinical, behavioural, and social factors that characterise these unique stages of life [3]. Pregnancy increases the vulnerability to climate-sensitive infectious diseases, particularly vector-borne diseases [3]. Infants and children also bear the greater burden climate-related disease (in terms of malnutrition, diarrheal disease, and malaria) given their immature immune systems, impaired thermoregulation, and lack of autonomy [2–6]. Moreover, women and children are often at greater risk of reduced survival and recovery in the aftermath of disasters, particularly when access to care is disrupted, and may suffer from more severe mental health consequences, with potentially long-lasting effects [3,7,8]. Climate change impacts maternal and newborn health (MNH) through a complex network of interconnected pathways that are exacerbated by geography, poverty, and women’s lack of empowerment [3] and that lead to overall amplification of existing health disparities [5].

Air pollution is closely linked to climate change. The main driver of climate change is fossil fuel combustion, which is also the major cause of outdoor air pollution. The simultaneous occurrence of air pollution, heat, and other climate-related changes has led to worse air quality. Reductions in greenhouse gas emissions have had immediate benefits to health through reduced exposure to short-lived air pollutants [9]. Climate change will also increase air pollution exposures from dust and wildfires, while changes in weather will affect air pollutant generation and dispersion.

There is a growing body of epidemiological evidence on the associations of climate hazards and related environmental exposures (such as air pollution) with health outcomes among pregnant women and newborns [10–12]. These findings have led to calls to action to protect MNH from the changing climate through both mitigation (reducing greenhouse gases) and adaptation (managing climate risks) [13–16]. Despite this, MNH is recognised and addressed only in some national adaptation plans. An analysis of 119 nationally determined contributions submitted between 2020 and 2022 showed few direct references to maternal health (n = 23) which have only acknowledged the effects of climate change, with very few mentions of adaptation efforts to address the impacts [17].

Many countries are developing policy goals and targets for adaptation and are recognising the need for aligning them with climate justice to protect the populations in vulnerable conditions [3]. However, clear interventions to address climate change impacts on these populations are rarely proposed or elaborated [17]. This makes efforts to document the associations between climate-related hazards and MNH outcomes a key step in the justification of the allocation of resources towards adaptation responses that are tailored for pregnant and postpartum women and newborn.

With this review, we sought to identify existing systematic and scoping reviews of the effects of climate hazards and air pollution on MNH, in order to map and describe the current landscape of available epidemiological evidence on key climate risks on MNH.

## METHODS

### Search strategy

We searched Medline (via Ovid) on 6 February 2023 for systematic and scoping reviews on the impact of different climate hazards and air pollution on MNH outcomes. We designed the search strategy using MeSH terms and keywords related to 'climate/air pollution hazards,' 'maternal health,' and 'newborn health,' combining synonyms and related terms for each concept using the Boolean operator 'OR' and all three searches using 'AND' (Table S1 in the **Online Supplementary Document**). Aside from this search, we sent a request to MNH researchers asking them to identify existing reviews. We also searched the reference lists of relevant studies to identify other relevant literature.

### Inclusion and exclusion criteria

We structured the search to identify systematic and scoping reviews published between 1 January 2010 up to 6 February 2023, with the former date reflecting the signing of the Cancun Agreements at the United Nations Climate Change Conference. We set no restrictions on geographical region or language. The reviews could have focussed on quantitative or qualitative studies.

To be selected, reviews had to include a population of pregnant and/or postpartum women (including lactating women) and/or newborns (0–28 days of age), and they had to have assessed the impact of the following climate or air pollution hazards on MNH outcomes:

− High temperatures and hot seasons;
− Ambient air pollution (AAP) originating from both anthropogenic (fossil fuel combustion) and natural (wildfires, dust storms, etc.) sources of emissions;
− Disasters (hydro-meteorological events);
− Water quality and accessibility;
− Climate sensitive food insecurity and changes in dietary patterns;
− Climate sensitive infectious diseases.

We excluded any studies that:

− Were not systematic in methodology, i.e. narrative reviews, literature reviews without clear methods;
− Did not investigate at least one of the above mentioned climate hazards;
− Focussed only on chemical contaminants, tobacco smoke, dampness/mould, radioactivity, solid waste;
− Focussed only on geophysical or man-made disasters;
− Described the effects of food insecurity or changes in dietary patterns on MNH without mentioning or considering the direct or indirect relationship between climate change (including climate-related shocks) and food insecurity;
− Described the effects of infectious diseases on MNH without mentioning or considering the direct or indirect impact of climate change on infection distribution, transmission, infestation, or illness.

### Study selection

We imported all identified references into a reference manager (EPPI-Reviewer, version 6 (EPPI Centre, London, UK)) where we removed any duplicates. Two authors (FC and AP) screened titles and abstracts; one author (FC) conducted full text screening; and a randomly selected 10% of excluded references were double screened by another author (AP) for quality assurance (agreement rate between the two authors for the title/abstract screening was 94%). Any differences were resolved by discussion.

### Data extraction, charting, and synthesis

We piloted a data extraction form in Microsoft Excel, version 16.82 (Microsoft Corporation, Redmond, Washington, USA) for 10 studies and then adapted it with minor changes made to the categorisation of climate hazards to ensure proper capture of the wide variety of climate hazards presented across the studies. The final data extraction form (Table S2 in the **Online Supplementary Document**) queried data on the authors; year of publication; review aim; type of review; type of climate hazard and/or air pollutant measured or recorded (where possible, with specification of each type of climate hazard and/or pollutant investigated); type of MNH outcomes assessed (with specification, where possible, of the number of included studies investigating each outcome); number of included studies per systematic review; key findings; and additional information, including assessment of risk of bias.

We then synthesised the findings by first providing a descriptive summary and overview of the characteristics of the systematic reviews included in the report, after which we performed a narrative synthesis of the described MNH outcomes associated with the different climate hazards and/or air pollutants. As our objective was to map and describe the literature and epidemiological landscape, we did not conduct any type of quality assessment of the included reviews.

We followed the PRISMA-ScR guidelines in reporting our findings [18].

## RESULTS

**Figure 1:**
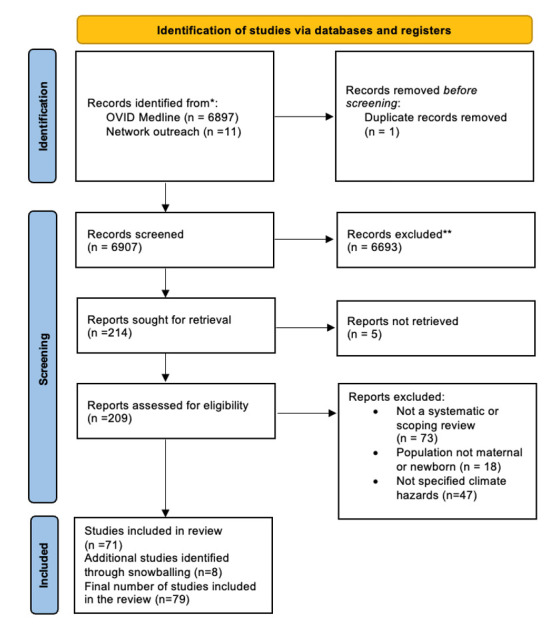
Study selection process.

We identified 6897 records from the database search and an additional 11 from our network outreach. After removing one duplicate, we screened the titles and abstracts of 6907 references, resulting in 214 potential records for inclusion. We could not retrieve the full text of five records, so we conducted full text screening for 209 records. After full text screening, 71 records were considered eligible for inclusion. We then identified an additional eight records by checking the references of the included studies (‘backward snowballing technique’), resulting in a final sample of 79 systematic and scooping reviews (**Figure 1**; Table S3 of the **Online Supplementary Document**).

### Characteristics of the included reviews

Most of the included reviews focussed on air pollution (n = 47, 59%), heat (n = 24/79, 30%), and flood/storm disasters (n = 7, 9%). We found only one review on water availability and quality and one assessing food insecurity in relation to climate change; no systematic reviews discussed climate-sensitive infectious diseases in our target groups in relation to the changing climate. One systematic review focussed on both heat and air pollution exposures and is therefore counted in the total number of reviews for each category.

A third of the included reviews featured a meta-analysis of the outcomes (n = 30, 38%). The majority of these (n/N = 24/30) investigate air pollution exposures. The remaining studies from the overall included sample were five scoping reviews (6%), two umbrella reviews (2%), one integrative review (1%), and one meta-ethnographic review (1%). In terms of year of publication, most of the 79 included reviews were recent, with 60 (76%) being published from 2015 onwards.

Quality appraisal of the included studies was not conducted in over a third of the 79 included reviews (n = 30, 38%). More than half of the reviews focussing on heat/seasonality (n/N = 13/24, 54%) and most of those focussing on disasters (n/N = 5/7, 71%) did not include a quality appraisal. Meanwhile, quality appraisal was conducted in more than two-thirds (n/N = 36/47, 77%) of the reviews investigating air pollution.

### Key findings by climate or air pollution hazard

**Table 1** summarises the range of MNH outcomes showing a positive association with respect to each climate hazard. Preterm birth was the most commonly investigated health outcome, mentioned in 30/79 (38%) of the included reviews. A description of key findings from each included review can be found in Table S4 in the **Online Supplementary Document**.

**Table 1:** Overview of identified MNH outcomes, categorised by climate hazard

Category of hazard	Associated maternal health outcomes	Associated foetal and perinatal health outcomes	Associated newborn health outcomes
High temperatures	Hypertensive disorders of pregnancy, gestational diabetes, mental health, access to health services	Miscarriage, stillbirth, congenital anomalies, preterm birth	Low birth weight, small-for-gestational age, hospitalisation, morbidity, mortality, sudden infant death syndrome, newborn feeding practices*
Ambient air pollution	Hypertensive disorders of pregnancy, gestational diabetes, mental health, access to health services	Miscarriage, stillbirth, intrauterine growth restriction, congenital anomalies, preterm birth	Low birth weight, small-for-gestational age, hospitalisation, morbidity, mortality, feeding practices
Disasters (hydro-meteorological)	Mental health, mortality	Miscarriage, preterm birth, mortality	Low birth weight, mortality, morbidity later in life, feeding practices
Water quality and accessibility	Hypertensive disorders of pregnancy	Not documented	Not documented
Climate sensitive food insecurity and dietary patterns	Mental health	Not documented	Not documented
Climate sensitive infectious diseases	Not documented	Not documented	Not documented

*Sudden infant death syndrome includes deaths after 28 days of age.

### High temperatures and hot seasons

We identified 24 reviews on the association of exposure to high temperatures and/or hot seasons with maternal, fatal, and perinatal and newborn health outcomes [19–42] (Table S5 in the **Online Supplementary Document**).

Ten reviews investigated the maternal health outcomes [19,20,27,28,30,31,33,37,39,42], with three finding evidence of an association between heat exposure and hypertensive disorders of pregnancy [19,27,33]. The meta-analysis by Beltran et al. [19] including 530 160 births showed an increased risk of pre-eclampsia for women with conception during the hottest months of the year (pooled relative risk (RR) = 1.25; 95% confidence interval (CI) = 1.10–1.42).

Gestational diabetes seemed to be associated with warmer seasons of the year, but there was no evidence regarding the effect of heat exposure. Koshhali et al. [37] identified a seasonal pattern for the diagnosis of gestational diabetes, with peaks in the warmer seasons, when the odds of being diagnosed increase by 12% (pooled odds ratio (OR) = 1.12; 95% CI = 1.03–1.21).

In a scoping review of qualitative studies, Aberese-Ako et al. [42] investigated access to maternal health services in relation to heat exposure; they synthesised factors that motivate or demotivate pregnant women in sub-Saharan Africa to access malaria interventions. Heat and warm weather emerged as important demotivating themes, particularly when coupled with the need to walk long distances to reach the health facility.

In the 14 reviews that investigated foetal and perinatal health [19–27,31,32,34,38], preterm birth was the most investigated outcome, with evidence of an association with exposure to extreme heat emerging from 12 reviews [19–23,26,27,31,32,34,35,39]. The systematic review and meta-analysis by Chersich et al [23] found evidence of a 16% increase in the odds of preterm birth during a heat wave compared to non-heat wave days (95% CI = 1.10–2.33), as well as an average increase of the odds of preterm birth of 1.05 for each 1°C increase in temperature (95% CI = 1.03–1.07).

Stillbirth also appeared to be linked to heat exposures, with eight systematic reviews confirming evidence of an association [21–24,26,27,32]. However, we identified fewer reviews focussing on other outcomes, such as miscarriage and congenital anomalies, with only one investigating miscarriage in relation to heat exposure and four reviews of epidemiological studies investigating congenital anomalies in relation to ambient heat, with mixed results.

Fifteen reviews assessed newborn health outcomes [19-23,26,27,29,31,32,35,36,39–41]. The strongest evidence emerged in favour of an association between exposure to heat and birth weight variations across 10 reviews [19,21–23,26,27,31,32,35], with lower birth weight more commonly observed in the warmer months of the year or following exposure to a heat wave.

According to five reviews [22,27,36,40,41], heat was also associated with increased risk of hospitalisations of newborns and infants. The relationship between heat and newborn/infant mortality was less studied, with only 2 systematic reviews [36,40] showing evidence of an increased mortality risk.

Lastly, only the review by Edney et al. [29] explored association between feeding practices and heat were the object of only one review, with a focus on low-and-middle income countries. They found that while exclusively breastfed infants appear to maintain normal hydration levels under hot conditions, feeding practices tend to be negatively affected by hot weather conditions through various pathways, including beliefs that infants may require supplementary liquids or increased seasonal demands on women’s time [29].

### Ambient air pollution

We identified 47 reviews documenting the association of exposure to AAP with maternal, foetal, and perinatal and newborn health outcomes [35,43–88] (Table S6 of the **Online Supplementary Document**).

The included reviews focussed on AAP originating from both anthropogenic (fossil fuel combustion) and natural (wildfires, dust storms, etc.) sources of emission. The types of air pollution identified across the included reviews represented the major health damaging pollutants according to the 2021 WHO Air Quality Guidelines [89]. Only 4/47 reviews investigated wildfire smoke exposures [51,54,55,88] and only one study focussed on dust storms [53].

With regards to maternal health outcomes, four reviews [45,47,60,64] found evidence of an association between AAP exposure and hypertensive disorders of pregnancy, while the evidence for gestational diabetes appeared less conclusive. The meta-analysis conducted by Pedersen et al. [45] found evidence of a 47% increase in the odds of hypertensive disorders of pregnancy per 5 μg/m3 increment of fine particulate matter (PM_2.5_), while increments of 10 μg/m3 of nitrogen dioxide (NO_2_) or coarse particulate matter (PM_10_) were associated with 23% and 11% increased odds of hypertensive disorders during pregnancy, respectively. In the review by Markozannes et al. [64], a 10 μg/m increase of PM_2.5_ levels during the third trimester was associated with an increased risk for hypertension in pregnancy (OR = 2.177, 95% CI = 1.710–2.773).

From the 32 reviews that investigated foetal and perinatal health [35,43,44,48–54,56–59,61,62,66,70–72,74,75,77–81,83–85,87,88], preterm birth was the most investigated outcome, with very good evidence of an association with prenatal exposure to air pollution emerging from 15 systematic reviews [35,43,50,51,53,56–58,70–72,79,84,85,87]. Ghosh et al. [87] performed a Global Burden of Disease Study and determined that 35.7% of global preterm births were attributable to total PM_2.5_ exposure, equivalent to 5 870 103 newborns in 2019 (meta-analysis coefficient of 12% increase in the risk of preterm birth per 10 μg/m3 increment in ambient PM_2.5_.) The evidence of ozone effects on birth outcomes was weaker. In the meta-analysis by Klepac et al. [79], whole pregnancy exposure to ozone (O_3_) was associated with a 3% increase in the odds of preterm birth per 10 ppb increment in O_3_.

Five reviews [31,40,67,70,73] explored the relationship between stillbirth and air pollution, with a focus on particulate matter; they all found evidence of an association, although the results presented by Siddika et al. [77] did not show statistical significance. Zhang et al. [44] noticed an association between PM_2.5_ exposure throughout the entire pregnancy and 10% increased odds of stillbirth, while Xie et al. [74] found a 15% increase in the odds of stillbirth per 10 μg/m3 increments of PM_2.5_. Air pollution appeared to be linked also to congenital anomalies in seven reviews [52,58,62,71,74,77,79], with cardiac congenital anomalies representing the most reported defects in association with the exposure. Exposure to air pollution, specifically particulate matter, was associated with increased risk of miscarriage in four systematic reviews [59,61,71,80].

Twenty-eight reviews [35,43,48,51–58,63–65,67–72,76,79,82,84,86–88] assessed newborn health outcomes, with most investigating the association between air pollution and birth weight variations. Lower birth weights following exposure to AAP were observed across 17 reviews [35,52–54,56–58,64,67,68,70–72,76,79,84,87]. The meta-regression by Ghosh et al. [87] found evidence of 22 g (95% uncertainty interval (UI) = 12–32) lower birth weight per 10 μg/m3 increment in ambient PM_2.5_ and estimated that 15.6% (95% UI = 15.6–15.7) of all newborns born weighing less than 2500 g globally was attributable to fine particulate matter exposure (equivalent to 2.8 million newborns in 2019). Eight reviews [42,52,56–58,64,79,84] found an association between exposure to AAP and small-for-gestational age newborns.

There is much less evidence regarding the impact of outdoor air pollution on newborn/infant mortality. One review addresses exposure to AAP (PM 2.5 and PM 10) NO and SO2) [84] and one review address exposure to wildfire smoke [54]. Morbidity outcomes in relation to air pollution exposure were assessed across seven reviews [55,56,63,65,69,82,86] with evidence emerging for increased odds of childhood wheezing or asthma in children exposed prenatally to air pollution [63] and up to a 32% increase in the odds of autism spectrum disorder in children exposed prenatally to fine particulate matter [65]. Henry et al. [55] found evidence of increased emergency department visits in children aged 0–18 years following exposure to wildfire smoke.

The impact of air pollution on feeding practices was assessed in a review conducted by Evans et al. [88]. The review included only one qualitative study exploring the relationship between exposure to pollution due to wildfire smoke, reporting that in postpartum women the exposure appears to be associated with reduced access to lactation support and lack of safe and private places, with declining breastfeeding rates during and after an evacuation.

### Flood/storm disasters

We identified seven reviews on the association between exposure to hydro-meteorological disasters, as defined by the 2020 WHO Glossary of Health Emergency and Disaster Risk Management Terminology [90] and maternal, foetal, and perinatal and newborn health outcomes [91–97]. The types of hydro-meteorological disasters identified were floods, hurricanes, and windstorms, although most of the included systematic reviews also assessed MNH outcomes in relation to other types of disasters (such as geophysical or technological disasters).

With regards to maternal health outcomes, two systematic reviews [91,92] found evidence of an association between experiencing a hurricane and the development of mental health conditions (including depression and posttraumatic stress disorder), while this association was noted in one systematic review investigating floods [96]. Only one systematic review [96] assessed maternal mortality following flooding, with evidence of an association between the disastrous event and an increased risk of maternal death.

Five reviews investigated foetal and perinatal health outcomes [91,92,94–96]. Zotti et al. [92] found evidence of an association between pregnant women experiencing a hurricanes and preterm birth, while this relationship was less consistent in the other systematic reviews [91,92,95]. Three systematic reviews found limited evidence of an association between foetal distress and hurricanes, although the finding was supported by only one review in each case [91,92,94]. Mallet et al. [96] observed increased risk of perinatal mortality following a flood, as well as an association between floods and increased risk of miscarriage, which also noted by Harville et al. [91].

Four reviews [91,92,94,95] assessed newborn health outcomes. Two showed an association between modifications in birth weight and exposure to a hurricane or a flood [91,92]. Mallet at al. [96] found evidence of an increased risk of newborn mortality as described within the findings related to under-five mortality following a flood. Hwang et al. [95] explored feeding practices and showed that mothers reported challenges in maintaining exclusive breastfeeding during disasters, due to lack of privacy, stress/exhaustion, limited fluid/nutritious intake.

Three reviews [92,93,96] investigated a range of short- and long-term effects on the children of maternal stress (caused by a flood, hurricane, or windstorm), all finding evidence of a relationship between disaster-related maternal stress and impacts on the mental and physical health of their children. In their meta-regression analysis, Lafortune et al. [94] observed a significantly positive overall association between prenatal maternal stress and offspring motor outcomes in flood related effect sizes (r = 0.0741; standard error (SE) = 0.0153, *P* > 0.0001) and a significantly positive overall association between prenatal maternal stress and offspring behavioural outcomes in flood related effect sizes (r = 0.0752; SE = 0.0170, *P* > 0.0001).

### Findings for other climate hazards

We identified one review documenting the relationship between water quality and maternal health outcomes [98] and none investigating the impact on foetal, perinatal and newborn health outcomes. The existing systematic review looked at epidemiological studies on the association between sodium in drinking water (associated with salinization) and changes in maternal blood pressure or hypertension [98]; the authors found weak evidence of an association due to the small number of studies with little sample sizes.

Only one review assessed the evidence of the association between food insecurity and maternal health outcomes [99], while none focussed on perinatal and/or newborn health. This aforementioned review by Trudell et al. [99] focussed on studies from the African continent and investigated the relationship between food insecurity and mental health which, according to the authors, is also significantly impacted by seasonal trends. The authors also noted how exposure to food insecurity appeared to be particularly associated with mental health issues among mothers (including depression and anxiety) and highlighted seasonality as a significant mediator of this association.

We identified no reviews investigating the impact of climate-sensitive infectious diseases and MNH outcomes.

## DISCUSSION

Our review describes the impacts of climate hazards on a wide range of MNH outcomes as identified in multiple systematic reviews and scoping reviews with the aim of providing an overview of the literature investigating MNH and climate change.

Many of the included reviews highlight links between high temperatures and adverse birth outcomes such as preterm birth. Although the mechanisms through which heat can trigger adverse MNH outcomes such as preterm birth have not been fully explained, a recent review of evidence from an expert group hypothesises that reduced placental blood flow, oxidative stress, and release of inflammatory markers could be involved [100]. While causal pathways have yet to be determined, the epidemiological evidence seems to strongly suggest that exposure to high temperatures increases the risk of preterm birth and stillbirth. Meanwhile, evidence regarding heat impacts on maternal health and newborn health is lacking, and none of the included reviews investigated the effect of high temperatures on maternal mortality. Heat exposure during early pregnancy appears to be associated to pre-eclampsia development, while the evidence of an impact of heat on gestational diabetes is limited.

The majority of the studies on heat and birth outcomes were conducted in high-income countries. As the frequency and intensity of exposure to heat waves are expected to increase globally, there is reason to assume that the existing burden related to adverse outcomes in pregnant women and newborns will likely rise as well, which is particularly concerning for countries that already have a high burden of maternal and neonatal mortality [3].

Moreover, most of the included reviews provide evidence of the impacts of AAP on MNH and highlight the detrimental effects of various pollutants, with compelling evidence on the association between fine particulate matter and adverse pregnancy and birth outcomes. This evidence supports actions to reduce fossil fuel combustion and transport emissions, as well as promote clean energy [3,101]. Such climate change mitigation policies would provide a direct and immediate benefits to maternal and newborn health.

Vulnerability to climate change varies not just across time and location, but also across individuals within communities [3]. Specific characteristics, such as age, gender, socioeconomic status, working conditions, and access to livelihood assets, mediate the impacts of climate-related exposures. The review by Bekkar et al [35] showed a disproportionate effect of air pollution and heat on pregnant women with certain medical conditions or specific race/ethnicities. Gendered vulnerabilities and impacts have been previously reported in relation to disasters, as previous reports documented women’s increased likelihood to die compared to men during some disasters [102,103], suggesting that conditions during and after a disaster often reflect and reinforce gender inequalities. However, the evidence on this phenomenon is still quite limited. The included systematic reviews focussed on exposure to hydro-meteorological disasters and describe a limited range of MNH outcomes associated to the exposure. Further, maternal stress was only considered in the context of outcomes in children, and specific evidence on maternal morbidity and mortality is limited.

Potential mechanisms through which short- and long-term effects of disasters on MNH are mediated by social, behavioural, and environmental effects have been conceptualised by Harville et al. [104]. In the short-term, physical trauma, adverse environmental exposures, and poor quality/insecure housing play a key role; in the long-term, relocation, changes in family functioning, and negative economic effects seem to have a greater effect. These aspects of disaster exposure can lead to lack of access to health care; increased stress and negative mental health outcomes; and negative behavioural changes, especially when populations are displaced. A recent multi-country analysis found evidence of increased risk of miscarriage among women experiencing gestational flood exposure in developing countries, suggesting how disparities in maternal health may be exacerbated during disasters [105].

Vulnerability to climate change can be experienced through direct exposure to extreme weather such as heat hazards or disasters, or indirectly through water and sanitation systems and food systems (which are also affected by disasters). These indirect effects are more difficult to study, as they require information on population-wide environmental exposure. We only identified one systematic review investigating the relationship between water quality (in terms of salinization) and maternal health. Stresses and shocks associated with climate change can lead to household food insecurity, particularly among women and girls [106]. This is particularly problematic in combination with the increased nutritional needs during pregnancy [3]. Maternal malnutrition is well known risk factor for serious pregnancy and birth complications [107]. We identified one systematic review that highlighted the relationship between food insecurity and greater risk of depression and anxiety among pregnant women in Africa [99], an interesting finding on an additional risk posed by malnutrition during pregnancy.

We did not identify any reviews investigating the impact of climate on infectious diseases on MNH. This represents a relevant gap, as climate change will increase risks of transmission for a range of diseases, particularly those transmitted by vectors [3]. Many vector-borne diseases, including malaria and arboviruses such as Zika and dengue, are particularly problematic for pregnant women [108]. For example, pregnant women with malaria are three times more likely to suffer from severe disease compared to their non-pregnant counterparts [103]. Climate change is projected to increase the seasonal transmission of malaria, as well as expand its range in the East African highlands and may make vector borne diseases hard to control.

Our review demonstrates that, although there is an abundance of epidemiological literature on climate change and MNH, research gaps remains. Most systematic reviews included in this report present evidence from high-income settings; relatedly, large epidemiological studies are more likely to be conducted in high-income countries. Therefore, evidence using robust data sources from low-income countries is needed. The findings of a recent study conducted across 14 low-and-middle income countries focussed on heat exposure by linking globally gridded meteorological data with spatially and temporally resolved Demographic and Health Survey data on adverse birth outcomes [108]. The research team found that experiencing higher temperatures and smaller diurnal temperature range during the last week before birth increased the risk of preterm birth and stillbirth [109].

The systematic and scoping reviews included in this report do not describe the impact of climate hazards on groups with vulnerable conditions, such as pregnant women who misuse alcohol or drugs; homeless women; women living in informal settlements; pregnant women who are recent migrants; asylum seekers; individuals with difficulties reading; pregnant women who experience domestic abuse; and pregnant women living with HIV or other chronic diseases. We also did not identify systematic reviews addressing occupational heat risks in pregnant and lactating women, including in both formal and informal sectors.

The effects of climate change on access to care and delivery of care were investigated only in a few reviews, and we identified no information on the impact on the quality of care. There is a need to better understand the impacts of climate change on MNH service delivery and quality of care across all categories of climate hazards and across diverse settings.

While this review summarises a wide range of potential negative impacts of different climate hazards on MNH, limitations need to be acknowledged. We searched only one database and identified only English language articles. Moreover, we were unable to retrieve the full text for five systematic reviews; even with the support of a librarian; these reviews were in Chinese and the principal author emails were not available. Therefore, we may have missed potentially relevant systematic reviews. We did, however, reach out to networks of experts in the climate change and MNH fields to ensure inclusion of relevant systematic reviews that might not have been captured by the search.

Further, we did not conduct quality assessment of the reviews, as our aim was to provide an overview of the current state of evidence and knowledge gaps. While such an assessment of the reviews would assess if the reviews were conducted with quality processes, it still does not relay the quality of the underlying primary studies and the confidence in those findings. We did note that over one third of the included systematic reviews did not feature any critical appraisal, suggesting that particularly the reviews addressing heat and disaster risks may be of lesser quality. However, we also included scoping reviews which normally may not assess the quality of included studies. More generally, challenges exist in synthesising and interpreting studies that examine impacts of environmental exposures on maternal, perinatal, and newborn health outcomes due to the heterogeneity across studies in definition and assessment of exposures, discrepancies in lag measures, and potential for confounding and effect modification. For example, studies that only look at associations between season and maternal health suffer from significant seasonal confounding, which would be better addressed through time series analyses. Studies that investigate extreme heat often use different measures of temperature and heat. Additionally, inconsistencies in the definitions of MNH outcomes across the different included reviews also need to be acknowledged. These aspects would also be important to consider in the quality of reviews addressing climate change impacts on health [110].

Effective policy responses to climate change on maternal and newborn health require integrating diverse mitigation and adaptation measures that address the unique needs of MNH. Rigorous research is required to improve our understanding of the impact of climate change on MNH and to inform development and implementation of strategies, policies, and programmes that allow to proactively prepare for and manage increasing threats posed by a wide range of climate hazards, including developing sustainable options for adaptation that can be tested and subsequently scaled-up.

## CONCLUSIONS

This review brings together the findings of systematic and scoping reviews covering five categories of climate hazards. This broad approach provides an initial and broad view of climate risks to maternal, perinatal, and newborn health and highlights the potential of the climate crisis to undermine recent improvements in maternal and newborn mortality and morbidity. We hope our findings will support the MNH community in better understanding potential MNH risks in different climate hazards, thereby allowing them to better engage in discussions on how MNH needs can be protected in different events. There is a need to strengthen the evidence base of primary research, particularly ensuring increased studies from low- and middle-income countries, as well as to strengthen review methods to consider MNH and climate epidemiology needs. There is also a notable lack of evidence for some climate hazards and for many maternal, perinatal, and newborn outcomes. Nonetheless, several studies repeatedly show associations for the impacts of heat and air pollutants on birth outcomes, particularly preterm birth. It is time for policy action and financing to consider the specific needs of MNH in climate change hazards.

## Additional material


Online Supplementary Document
