# Supplementary material for: Climate change, air pollution and maternal and newborn health: An overview of reviews of health outcomes
Source: J Glob Health. 2024 May 24;14:04128. doi: 10.7189/jogh.14.04128 (PMC11117177; doi:10.7189/jogh.14.04128)
Supplement: Online Supplementary Document [file jogh-14-04128-s001.pdf]

Online Supplementary Document S1

Table S1. Search Strategy.

#	Query	Results from 6 Feb 2023
1	exp Pregnancy/	992,138
2	exp Maternal Mortality/ or exp Maternal Health/	13,292
3	exp Pregnancy Complications/	469,099
4	exp preconception care/ or exp prenatal care/	34,000
5	exp Delivery, Obstetric/ or exp Labor, Obstetric/ or exp Cesarean Section/	122,681
6	exp Puerperal Disorders/ or exp Postpartum Period/	106,527
7	exp parturition/ or exp placentation/ or exp pregnancy in adolescence/ or exp pregnancy outcome/ or exp abortion, spontaneous/	129,651
8	exp Infant, Newborn/	666,186
9	exp Stillbirth/ or exp Premature Birth/	25,470
10	exp Infant, Newborn, Diseases/	188,986
11	exp Breast Feeding/	43,215
12	exp Infant Mortality/	31,616
13	1 or 2 or 3 or 4 or 5 or 6 or 7 or 8 or 9 or 10 or 11 or 12	1,597,434
14	exp Climate/ or exp Climate Change/	181,273
15	exp Hot Temperature/	124,351
16	exp Weather/ or exp Extreme Weather/ or exp Extreme Hot Weather/	572,048
17	exp Air Pollution/	68,827
18	exp Particulate Matter/	77,119

#	Query	Results from 6 Feb 2023
19	exp Carbon Dioxide/	96,785
20	exp Ozone/	17,326
21	exp Sulfur Dioxide/ or exp Air Pollutants/ or exp Environmental Exposure/	405,360
22	exp Climatic Processes/	71,445
23	exp Air Movements/	8,513
24	exp Disasters/ or exp Droughts/	98,363
25	exp Floods/	3,788
26	exp Salinity/ or exp Water Pollutants, Chemical/	135,321
27	exp Landslides/	255
28	exp Tornadoes/	228
29	exp Wildfires/	1,065
30	exp Sanitation/	114,299
31	exp Water Supply/ or exp Water/ or exp Water Quality/	246,417
32	exp food supply/ or exp famine/ or exp food insecurity/ or exp food security/	16,711
33	exp communicable diseases/ or exp pregnancy complications, infectious/ or exp vector borne diseases/ or exp virus diseases/ or exp waterborne diseases/ or exp zoonoses/	1,491,128
34	14 or 15 or 16 or 17 or 18 or 19 or 20 or 21 or 22 or 23 or 24 or 25 or 26 or 27 or 28 or 29 or 30 or 31 or 32 or 33	3,044,635
35	13 and 34	171,303
36	limit 35 to ("review articles" and humans and yr="2010 -Current")	6,897

Online Supplementary Document S2

Table S2. Template of the data extraction sheet.

Name of first author and year of publication	Review type	Review aim	Climate hazards	MNH outcomes	Number of included studies	Key findings	Risk of bias conducted

Online Supplementary Document S3

Table S3. Reasons for exclusion at full text screening stage.

Name of first author (year of publication)	Reasons for exclusion at full text screening stage
Adachi et al. (2018); Adam et al. (2011); Balise et al.(2016); Bastian et al. (2022); Benova L (2014); Bharatraj et al. (2018); Bihaqi et al. (2019); Brent et al. (2014); Brooks et al. (2020); Brown et al. (2011); Brown et al. (2012); Burris et al. (2016); Caserta et al. (2011); Cortés-Albornoz et al. (2021); Davis et al. (2019); Dìaz-Anzaldùa et al (2013); Dutta et al. (2022); Ferguson et al. (2013); Giudice et al. (2021); Grippo A (2017) ;Haruty et al. (2016); Hassan et al. (2019); Hei et al (2014); Kahn et al. (2018); Kalkbrenner et al. (2014); Kappil et al. (2015); Kihal-Talantikite at al. (2017); Lee KK (2020); McCue et al. (2019); Misra P (2012); Nieuwenhuijsen et al. (2013); Patelarou E (2014) ; Pope DP (2010) ; Pratiti R (2020) ; Protano et al. (2012); Pun VC (2021) ; Rahman et al. (2016); Ralphe et al. (2018); Rodriguez-Soto et al. (2021); Sathyanarayana et al. (2012); Schoeters et al. (2011); Tchernitchin et al. (2018); Urgell-Lahuerta et al. (2021); Varshavsky et al. (2019); Wang et al. (2022); Wei et al. (2021); Woolley et al. (2022); Younger A (2022).	Excluded on exposure (not specified climate hazards)
Ades et al. (2020); Al-Gubory et al.(2014); Al-Gubory et al. (2016); Anderko et al. (2020); Andersen et al. (2016); Basilio et al. (2022); Bearblock et al. (2020); Boda et al. (2020); Brodie et al. (2021); Burris et al. (2011); Burris et al. (2017); Canales et al. (2015); Carlsten et al. (2012); Chersich et al. (2023); Collaco et al. (2021); Conry et al. (2022); Costa et al. (2019); Costa et al. (2020); Cumming et al. (2016); Erickson et al. (2014); Gentner et al. (2019); Ghazi et al. (2021); Gòmez-Roig et al. (2021); Gonzalez et al. (2022); Ha et al. (2022); Hertz-Picciotto et al. (2018); Kloog et al. (2019); Korten et al. (2017); Levy et al. (2015); Li et al. (2018); Martin et al. (2018); Mbuya et al. (2016); Miki et al. (2022); Miller et al. (2018); Morris et al. (2021); Murphy et al. (2021); Myhre et al. (2018); Nakstad et al. (2022); O'Rourke et al. (2017); Olson et al. (2019); Olson et al. (2020); Pace et al. (2021); Pacheco et al. (2020); Padula et al. (2020); Pandipati et al. (2022); Perera et al. (2011); Proietti et al. (2012); Ragusa et al. (2021); Rothschild et al. (2023); Saenen et al. (2019); Sealey et al. (2016); Segal et al. (2019); Sharpe et al. (2010); Sheffield et al. (2010); Shezi et al. (2020); Slama et al. (2013); Slama et al. (2013); Smarr et al. (2013); Soto-Martinez et al. (2010); Tan et al. (2017); Teng et al. (2016); Thyssen et al. (2015); Vallés et al.(2018); Veras et al. (2017); Veras et al. (2021); Vrijheid et al. (2012); Vrooman et al. (2016); Wang et al. (2020); Weisskopf et al. (2015);	Excluded on review design (not a systematic or scoping review)

Name of first author (year of publication)	Reasons for exclusion at full text screening stage
Westergaard et al. (2017); Whaibeh et al. (2022); Yi et al. (2022): Zhang et al. (2021).	
Bernabucci et al. (2013); Bowatte et al. (2014); Cumming et al. (2010); D’Angelo et al. (2021); Da Costa E Oliveira et al. (2019); Dutheil et al. (2021); Frolkis et al. (2013); Imbriani et al. (2021); Johnson et al. (2021); Mannucci et al. (2015); Morales-Suàrez et al. (2016); Nelson et al. (2017); Patelarou et al. (2015); Reid et al. (2016); Suades-Gonzalez et al. (2015); Vallianou et al. (2012); Vieira et al. (2015); Yang et al. (2017).	Excluded on population (not MNH*)

*MNH= Maternal and newborn health

Online Supplementary Document S4

Table S4a. Reviews on high temperatures and hot seasons and MNH outcomes (N=24).

Name of first author and year of publication	Review aim	Climate Hazards*	MNH Outcomes**	Number of included studies & Key Findings	Other Information
Strand LB (2011).	To systematically review the epidemiological evidence on seasonality of birth outcomes and the influence of ambient temperature through a narrative review approach.	-Seasonality.<br>-Temperature. (heat/cold).	-PTB = 14 studies (8 studies investigate PTB in relation to seasonality and 6 studies in relation to ambient temperatures)<br>-Stillbirth= 4 studies<br>-Birth weight= 18 studies (10 studies investigate birth weight in relation to seasonality and 8 studies in relation to ambient temperature)	32 studies included in the systematic review.<br><br>-Seasonal patterns may increase the risk of preterm birth (5/8 studies found increased incidence of preterm births during the summer).<br><br>-Seasonal patterns may increase the risk of stillbirths (3 /4 studies found increased incidence of stillbirth during the winter).<br><br>-Seasonal patterns may influence birth weight (lowest mean birth weights observed in summer and winter in 5/10 studies).<br><br>-Exposure to high ambient temperatures during pregnancy is associated with preterm births (4/6 studies).<br><br>-Inconsistent evidence with regards to ambient temperature exposure during pregnancy and birth weight.	Risk of bias: no evidence of quality assessment.
TePoel MRW (2011).	To systematically review the evidence on the relationship between seasonality and risk of	-Seasonality.	-Hypertensive disorders of pregnancy= 20 studies (14 conducted in non-tropical settings, 6 conducted in tropical settings)	20 studies included in the systematic review.<br><br>-Seasonal patterns in non-tropical settings may increase the rate of hypertensive disorders of pregnancy (highest rates of hypertensive disorders of pregnancy observed during the winter in 11/14 studies).	Risk of bias: no evidence of quality assessment.

Name of first author and year of publication	Review aim	Climate Hazards*	MNH Outcomes**	Number of included studies & Key Findings	Other Information
	pre-eclampsia and eclampsia through a narrative review approach.			-Seasonal patterns in tropical settings may increase the rate of hypertensive disorders of pregnancy (highest rates of hypertensive disorders of pregnancy observed during the wet and rainy season in 5/6 studies).	
Xu Z (2012).	To systematically assess the association between temperature and child (0-18 years) mortality and morbidity through a narrative review approach.	-Temperature (heat/cold)	-Child mortality= 12 studies<br>-Child morbidity=21 studies	33 studies included in the systematic review.<br><br>-Exposure to high temperatures is associated with increased risk of child mortality (9/10 studies), particularly in children under one year of age (4/10 studies).<br><br>-Exposure to high temperatures is associated with increased risk of all cause hospital admissions (2/20 studies), diarrhoea (5/20 studies), electrolyte imbalance (1/20 studies), respiratory disease (1/20 studies), hand foot and mouth disease (1/20 studies), infectious gastroenteritis (1/20 studies),	Risk of bias: no evidence of quality assessment.
Carolan-Olah M (2013).	To systematically examine the evidence of an association between high environmental temperatures and preterm birth through a narrative review approach.	-Temperature (heat).	-PTB=7 studies	7 papers included in the systematic review.<br><br>-Exposure to high ambient temperature during pregnancy is associated with preterm births (5/7 studies).	Risk of bias: Quality assessment conducted for all included studies.
Xu Z (2014).	To systematically assess the association between	-Temperature (heat waves)	-Child mortality=9 studies<br>-Child morbidity= 5 studies	12 studies included in the systematic review.<br><br>- Inconsistent evidence with regards to exposure to heat waves and increased risk of child mortality.	Risk of bias: no evidence of quality assessment.

Name of first author and year of publication	Review aim	Climate Hazards*	MNH Outcomes**	Number of included studies & Key Findings	Other Information
	temperature, specifically heat waves and child (0-18 years) mortality and morbidity through a narrative review approach.			-Exposure to heat waves is associated with increased risk of child hospital admissions (2/5 studies)	
Beltran AJ (2014).	To synthesize evidence on the effects of meteorology on pregnancy outcomes through a narrative review approach; to conduct a meta-analysis and present meta-effect size estimates.	-Seasonality.<br>-Temperature (heat/cold).<br>-Barometric pressure.	-Hypertensive disorders of pregnancy (gestational hypertension, pre-eclampsia, eclampsia) =35 studies.<br><br>-Length of gestation (median gestational length, preterm birth (PTB), onset of labour, premature rupture of membranes) =28 studies.<br><br>-Birth weight (mean birth weight, low birth weight (LBW) and small for gestational age (SGA)) =27 studies.	90 studies were included in the systematic review.<br><br>-A meta-analysis including 530,160 births (7 studies) showed an increased risk of pre-eclampsia for women with conception during the hottest months of the year (pooled relative risk (RR)=1.25, 95% CI (1.10, 1.42) for August).<br><br>-Exposure to high temperature during the month or in the days preceding birth is associated with decreased gestational length. A meta-analysis of 63,227,292 births (6 studies) showed peaks of preterm birth at the beginning of summer (pooled relative risk (RR)= 1.19, 95% CI (1.14, 1.23).<br><br>-Birth weights are lower for births occurring in winter and summer. A meta-analysis of 70,625,872 term and preterm births (6 studies) showed lowest birth weights in the months of December and July. Similar findings derived from a meta-analysis of 5,398,360 term births.	Systematic review and meta-analysis.<br><br>Risk of bias: no evidence of quality assessment.
Poursafa P (2015).	To synthesize the relationship	-Seasonality.<br>-Temperature (heat/cold).	-Hypertensive disorders of pregnancy (gestational hypertension, pre-	15 studies were included in the systematic review.	Risk of bias: no evidence of quality assessment.

Name of first author and year of publication	Review aim	Climate Hazards*	MNH Outcomes**	Number of included studies & Key Findings	Other Information
	between climate change and climate variability and pregnancy outcomes through a narrative review approach.		eclampsia, eclampsia) =7 studies.<br><br>-Congenital anomalies=1 study.<br><br>-PTB/ gestational length=5 studies.<br><br>-LBW=2 studies.<br><br>*Note: no definitions provided for MNH outcomes.*	-Exposure to high temperatures or low temperatures during pregnancy is associated with increased risk of preterm birth in 3/5 studies.<br><br>-Inconsistent evidence of association between season of delivery or conception and risk of developing pre-eclampsia.	
Megaw L (2017).	To systematically review the literature on the relationship between ultraviolet (UV) radiation and singleton pregnancy outcomes through a narrative review approach.	-Ultraviolet radiation.	-Hypertensive disorders of pregnancy = 2 studies<br>-Birth weight = 3 studies<br>-Gestational length (including PTB) = 2 studies	7 studies included in the systematic review.<br><br>-Inconsistent evidence with regards to the effect of UV radiation exposure on fetal growth.<br>-Increases in the annual average UV index associated with increased preterm birth (1 study, $p<0.0001$).<br>-Inconsistent evidence with regards to UV radiation exposure during pregnancy and development of hypertensive disorders of pregnancy.	Risk of bias: Quality assessment conducted for all included studies.
Kuehn L (2017).	To assess how extreme heat affects maternal, fetal and neonatal health., through a	-Temperature (heat and heat waves).	-Early term birth=6 studies<br><br>-PTB=17 studies<br><br>-LBW=5 studies<br><br>-Stillbirth=3 studies	28 studies included in the systematic review.<br><br>-High temperature exposure during pregnancy is associated with increased risk of preterm birth in 15/17 studies and increased risk of early term birth in 5/6 studies.	Risk of bias: no evidence of quality assessment.

Name of first author and year of publication	Review aim	Climate Hazards*	MNH Outcomes**	Number of included studies & Key Findings	Other Information
	narrative review approach.		-Newborn health= 2 studies<br><br>*Note: the definition of stillbirth varied across studies (stillbirth defined as fetal death after 12 weeks' gestation, or after 20 weeks' gestation or after 24 weeks' gestation).*	-High temperature exposure during pregnancy is associated with increased risk of low birth weight in 3/5 studies.<br>-High temperature exposure during pregnancy is associated with increased risk of stillbirth in 2/3 studies.<br>-Heat wave exposure (>42°C) is associated with increased NICU admissions (1 study).<br>-Evidence of effect modification within infant death rates during warm seasons (1 study).	
Zhang Y (2017).	To review epidemiological evidence on the relationship between temperature exposure during pregnancy and PTB, LBW and stillbirth through a narrative review approach. .	-Temperature (heat/cold).	-PTB/ gestational length=24 studies.<br><br>-Birthweight= 14 studies.<br><br>-Stillbirth=4 studies.<br><br>.	36 studies included in the systematic review.<br><br>-High temperature exposure during pregnancy is associated to decreased gestational length across different climate zones in 21/24 studies.<br><br>-High temperature exposure during pregnancy is associated to reduced birthweight in 12/14 studies.<br><br>-High temperature exposure during pregnancy is associated with increased risk of stillbirth in 4/4 studies.	Risk of bias: Quality assessment conducted for all included studies.
Ravanelli N (2018).	To determine the critical environmental and exposure limits for exercise and/or heat	-Temperature (heat).<br>-Humidity.	-Maternal core temperature response to land-based exercise= 8 studies<br>-Maternal core temperature response to	12 studies included in the systematic review.<br><br>-There is low risk of maternal core temperature exceeding 39°C during land-based exercise independent of gestational age and modality and at intensities up to 90% of maximum heart rate, by fit pregnant women for up to 35	Risk of bias: Quality assessment conducted for all included studies.

Name of first author and year of publication	Review aim	Climate Hazards*	MNH Outcomes**	Number of included studies & Key Findings	Other Information
	exposure during pregnancy and to assess whether thermoregulatory capacity (indicated by changes in core temperature) during exercise and/or heat exposure is altered throughout pregnancy using a best evidence synthesis approach to synthesis and rank evidence.		water immersion exercise=3 studies<br>-Maternal core temperature response to passive heating at rest= 2 studies<br><br>*Note: the teratogenic maternal core temperature threshold is estimated at 39°C.*	minutes, in ambient temperatures not exceeding 25°C and 45% relative humidity (level B evidence, 8/12 studies).<br>-There is low risk of maternal core temperature exceeding 38°C during water-based exercise up to 33.4°C, independent of gestational age (level C evidence, 3/12 studies).<br>-There is low risk of maternal core temperature exceeding 38°C during passive heat exposure to warm baths (40°C) or sauna use (70°C 15% relative humidity) for up to 20 minutes (level D evidence, 2/12 studies).<br>-A pattern of smaller changes in core temperature following land based or water-based exercise was observed with progression of pregnancy (7/12) suggesting an enhanced thermoregulatory capacity with progression of pregnancy.	
Chersich M (2020).	To assess whether exposure to high temperatures in pregnancy is associated with increased risk for stillbirth, PTB and LBW; to perform a meta-analysis.	-Temperature (heat, heat waves).	-PTB/gestational length=47 studies<br><br>-LBW=28 studies<br><br>-Stillbirth=8 studies	70 studies included in the systematic review.<br><br>-High temperature exposure during pregnancy is associated with increased risk of preterm birth (40/47 studies). In a meta-analysis of six studies the odds of preterm birth during a heatwave were 1.16-fold higher than on non-heatwave days 95%CI (1.10,2.33; I^2 :44.7%). The meta-analysis showed an average odd of a preterm birth increased by 1.05 for each 1°C increase in temperature 95%CI (1.03,1.07)<br><br>-High temperature exposure during pregnancy over a trimester or all of pregnancy is associated	Systematic review and meta-analysis.<br><br>Risk of bias: Quality assessment conducted for all included studies.

Name of first author and year of publication	Review aim	Climate Hazards*	MNH Outcomes**	Number of included studies & Key Findings	Other Information
				with low birth weight (10/28 studies), median OR= 1.09 IQR (1.04,1.47; range 1.01, 2.49) or with decreased birth weight (Median difference in birth weight at high versus low temperatures –25.5 gr (range –39.4, -15.0).<br><br>-High temperature exposure during pregnancy is associated to increased risk of stillbirths (8/8 studies). In a meta-analysis of 3 studies, the odds of stillbirth per 1°C temperature increase increased by 1.05-fold 95%CI (1.01,1.08) and in a meta-analysis of 3 studies the odds of stillbirth during exposure to high temperatures over a trimester or all of pregnancy were 3.39-fold higher 95%CI (2.33,4.96).	
Preston EV (2020).	To systematically review epidemiological studies evaluating the associations of climate factors with gestational diabetes and glycaemic outcomes during pregnancy through a narrative review approach.	-Seasonality.<br>-Temperature. (heat/cold).<br>-Humidity.	-GD diagnosis in relation to seasonality= 12 studies<br>-Blood glucose levels during pregnancy in relation to seasonality= 11 studies<br>-GD diagnosis in relation to temperature= 8 studies<br>-Blood glucose levels during pregnancy in relation to temperature= 8 studies<br><br>*Note: heterogeneity in method of GD screening/diagnosis across the included studies. Included studies either used a one-step approach (IADPSG, WHO modified) or a two-step approach (ADIPS, Carpenter and*	16 studies included in the systematic review.<br><br>-Higher incidence/prevalence of GD diagnosis during the summer and lower incidence during the winter (7/12 studies).<br><br>-Inconsistent evidence with regards to the association between blood glucose levels during pregnancy and seasonality.<br><br>-High temperature exposure on the day of the GD screening or in the days/weeks prior to the GD screening is associated with increased risk of GD diagnosis (5/8 studies) and increased blood glucose levels (7/ 8 studies).	Risk of bias: Quality assessment conducted for all included studies.

Name of first author and year of publication	Review aim	Climate Hazards*	MNH Outcomes**	Number of included studies & Key Findings	Other Information
			Coustan, NDDG, WHO modified).		
Bekkar B (2020).	To systematically assess the association between exposure to heat and air pollution to birth outcomes in the United States using Arksey and O'Malley's methodological framework for scoping reviews.	-Temperature (heat).<br>-Air pollution (PM 2.5, ozone).	-PTB= 29 studies (24 studies investigate PTB in relation to air pollution, 5 studies in relation to heat)<br>-LBW=32 studies (29 studies investigate LBW in relation to air pollution, 3 studies in relation to heat)<br>-Stillbirth= 7 studies (5 studies investigate stillbirth in relation to air pollution, 2 studies in relation to heat)<br><br>*Note: articles analysing non-US populations were excluded.*	68 studies included in the systematic review.<br><br>-Exposure to heat during pregnancy is associated with increased risk of preterm birth (4/5 studies; 0.8 million total births; increased risk median 15.8% range (9.0-22.0) for whole pregnancy heat exposure).<br><br>-Exposure to heat during pregnancy is associated with increased risk of low birth weight (3/3 studies; 2.7 million total births; increased risk median 31.0% range (13.0-49.0) for whole pregnancy heat exposure).<br><br>-Exposure to heat during pregnancy is associated with increased risk of stillbirth (2/2 studies; 0.2 million total births; increased risk median N/A; stillbirth risk increased 6% per 1°C increase the week before delivery during the warm season in 1 study).	Scoping review.<br><br>Risk of bias: no evidence of quality assessment.
Paterson SK (2020).	To systematically assess the association between exposure to heat and health outcomes of vulnerable populations in temperate climatic regions, with a	-Temperature (heat)	-PTB= 3 studies<br>-Birth weight= 1 study	15 studies included in the scoping review. 3 studies provided information for MNH outcomes.<br><br>-Exposure to high temperatures during pregnancy is associated with preterm births (2/3 studies).<br><br>-Exposure to high ambient temperature during pregnancy is associated with reduced birth weight (1 study).	Scoping review.<br><br>Risk of bias: no evidence of quality assessment.

Name of first author and year of publication	Review aim	Climate Hazards*	MNH Outcomes**	Number of included studies & Key Findings	Other Information
	focus on Ireland.				
Sexton J (2021).	To assess the association between ambient temperature exposure during pregnancy and stillbirth, through a narrative review approach.	-Temperature (heat/cold).	-Stillbirth=12 studies	12 studies included in the systematic review.<br><br>-High temperature exposure during pregnancy is associated to increased risk of stillbirth (12/12 studies).	Risk of bias: Quality assessment conducted for all included studies.
Haghighi MM (2021).	To assess the association between environmental heat exposure during pregnancy and congenital anomalies through a narrative review approach.	-Temperature (heat).	-Congenital anomalies= 13 studies (6 studies reported on cardiac defects, 3 on neural tube defects, 2 on orofacial cleft or cranial defects, 2 on hypothyroidism, 1 on ocular anomalies, 1 on renal anomalies, 1 on hypospadias, 1 on musculoskeletal defects, 1 on lethal anomalies).	13 studies included in the systematic review.<br><br>-High temperature exposure during pregnancy is associated with increased risk of cardiac anomalies in 3/6 studies.<br><br>-Inconsistent evidence of association between high temperature exposure during pregnancy and neural tube defects and orofacial cleft/ cranial defects.<br><br>-High temperature exposure during pregnancy is associated with decreased risk of hypothyroidism in 2/2 studies.	Risk of bias: no evidence of quality assessment.
Khoshhali M (2021).	To systematically assess whether seasonal variations and temperature is associated with increased	-Seasonality<br>-Temperature (heat)	-GD= 13 studies	13 studies included in the systematic review. 11 studies included in the meta-analysis.<br><br>- Seasonality of GD screening is associated with the risk of GD (pooled OR=1.12, 95%CI (1.03-1.21) I^2 : 94.4%%).). The meta-analysis demonstrated a positive and significant association between warmer seasons and GD prevalence.	Systematic review and meta-analysis.<br><br>Risk of bias: Quality assessment conducted for all included studies.

Name of first author and year of publication	Review aim	Climate Hazards*	MNH Outcomes**	Number of included studies & Key Findings	Other Information
	risk of gestational diabetes; to perform a meta-analysis.			-The relationship between temperature and prevalence of GD is less consistent.	
Syed S (2022).	To assess the effects of extreme heat on pregnancy outcomes, using Arksey and O'Malley's methodological framework for scoping reviews.	-Temperature (heat).	-PTB= 44 studies<br>-Birth weight (mean birth weight, LBW) = 21 studies<br>-Congenital anomalies = 11 studies<br>-Stillbirth= 10 studies<br><br>*Note: no definitions provided for MNH outcomes.*	84 studies included in the systematic review.<br><br>-High temperature exposure during pregnancy is associated with preterm birth (40/44 studies).<br><br>-High temperature exposure during pregnancy is associated with low birth weight (16/21 studies).<br><br>-High temperature exposure during pregnancy (mostly first trimester exposure) is associated with increased risk of congenital cardiac anomalies (5/11 studies).<br><br>-High temperature exposure during pregnancy is associated with increased risk of stillbirth (10/10 studies).	Scoping review.<br><br>Risk of bias: no evidence of quality assessment.
Dalugoda Y (2022).	To describe and summarize the current evidence of the association between elevated ambient temperature exposure during pregnancy and a range of	-Seasonality.<br>-Temperature (heat/ heatwaves).	-PTB=30 studies<br>-LBW=11 studies<br>-Stillbirth=9 studies<br>- Gestational diabetes (GD)=8 studies<br>-Congenital anomalies=6 studies<br>-Neonatal mortality=6 studies<br>-SGA=2 studies<br>-Miscarriage=2 studies<br>-Hypertensive disorders of pregnancy=2 studies<br>-Placental abruption= 2 studies	75 studies included in the systematic review.<br><br>-High temperature exposure during pregnancy is associated with preterm birth (23/30 studies).<br>-High temperature exposure during pregnancy is associated with low birth weight (5/11 studies).<br>-High temperature exposure during pregnancy is associated with increased risk of stillbirth (9/9 studies).<br>-High temperature exposure during pregnancy is associated with increased risk of gestational diabetes diagnosis, higher glucose levels and increased prevalence of gestational diabetes (8/8 studies).	Scoping review.<br><br>Risk of bias: no evidence of quality assessment.

Name of first author and year of publication	Review aim	Climate Hazards*	MNH Outcomes**	Number of included studies & Key Findings	Other Information
	adverse maternal, fetal and neonatal outcomes using the PRISMA extension methodology.		-Premature rupture of membranes= 2 studies<br>-INR value of newborns= 1 study<br>-Newborn telomer length= 1 study<br>-Bacteriuria in pregnancy=1 study<br>-Cardiovascular events during labour= 1 study<br>-Neonatal morbidity= 1 study<br>Reduced placental weight/volume= 1 study<br><br>*Note: no definitions provided for most MNH outcomes*	-High temperature exposure during pregnancy is associated with hypertensive disorders of pregnancy (2/2 studies).<br>-High temperature exposure during pregnancy is associated with increased risk of reduced placental weights and volume (1 study).<br>-High temperature exposure is associated with increased risk of higher INR values in newborns (1 study).<br>-Inconsistent evidence with regards to the association between high temperatures and neonatal mortality and morbidity.<br>-Inconsistent evidence with regards to the association between high temperatures and small-for-gestational age.<br>-Inconsistent evidence with regards to the association between high temperatures and congenital anomalies.<br>-Inconsistent evidence with regards to the association between high temperatures and miscarriage.	
Edney JM (2022).	To systematically investigate how hot weather conditions may affect hydration levels in exclusively breastfed infants and impact infant feeding practices in low-and-	-Temperature (heat).<br>-Humidity.	-Effects on hydration levels in exclusively breastfed infants= 9 studies<br>-Effects on feeding practices= 9 studies<br><br>*Note: there is no formal measurement methodology for dehydration in infants- a mix of measures has been used to assess dehydration including urine specific gravity (USG), urine osmolality (UO), urine*	18 studies included in the systematic review.<br><br>-Exclusively breastfed infants under hot conditions maintain normal hydration levels without concentrating urine to maximal levels (9/9 studies).<br>-Exclusively breastfed infants can maintain normal hydration levels in hot and humid conditions (4/9 studies).<br>-Feeding practices may be affected by temperatures through various pathways (including beliefs that infants require supplementary liquids or increased seasonal demands on woman's time).	Risk of bias: Quality assessment conducted for all included studies.

**Name of first author and year of publication**	**Review aim**	**Climate Hazards***	**MNH Outcomes****	**Number of included studies & Key Findings**	**Other Information**
	middle income countries through a narrative review approach.		*volume, total fluid intake, and infant weight change.*		
Lakhoo DP (2022).	To systematically explore the associations between exposure to high and low ambient temperatures and acute adverse outcomes in infants.	-Temperature (heat/cold)	-Infant mortality= 11 studies<br>-Infant morbidity=10 studies<br>-Other neonatal conditions= 2 studies	26 studies included in the systematic review.<br><br>-Exposure to high temperatures is associated with increased infant mortality (6/6 studies).<br><br>-Inconsistent evidence with regards to temperature and sudden infant death syndrome.<br><br>-Exposure to high temperatures is associated with increased hospital visits or admissions for all cause morbidity in infants and for heat related morbidity in newborns (4/4 studies).<br><br>-Exposure to high temperatures is associated with increased risk of higher INR (1 study) and of being referred for neonatal jaundice (1 study).	Risk of bias: Quality assessment conducted for all included studies.
Tung TH (2022).	To systematically assess the association between seasonal variation and postpartum depression; to perform a meta-analysis.	-Seasonality	-Postpartum depression (PPD)= 5 studies	5 studies included in the systematic review. 5 studies included in the meta-analysis.<br><br>-Season of delivery is associated with the risk of PPD, as women giving birth in spring, summer of autumn have a lower risk of PPD compared to those that give birth in winter (RR: 0.83, 95%CI (0.78-0.88)).	Systematic review and meta-analysis.<br><br>Risk of bias: Quality assessment conducted for all included studies.
Aberese-Ako M (2022).	To present the findings on the factors that motivate or demotivate	Temperature (heat)	Motivators to access malaria health services in pregnancy	24 studies included in the meta-ethnographic review.	Meta-ethnographic review.<br><br>Risk of bias: no evidence of quality assessment.

Name of first author and year of publication	Review aim	Climate Hazards*	MNH Outcomes**	Number of included studies & Key Findings	Other Information
	women from accessing malaria in pregnancy interventions in Sub-Saharan Africa, through a meta-ethnographic review approach.			-Warm weather demotivates pregnant women from using long-lasting insecticidal nets (5/24 studies).<br><br>-Warm weather, coupled with long distances, demotivates pregnant women from accessing antenatal care health services (3/24 studies).	

MNH: Maternal and newborn health. LMICs: low-and-middle income countries. PTB: preterm birth. LBW: low birth weight. GD: gestational diabetes. CHD: congenital heart disease. PPD: postpartum depression. GCT: glucose challenge test. OGTT: oral glucose challenge test. SGA: small for gestational age. INR: international normalized ratio. USG: urine specific gravity. UO: urine osmolality. UV: ultraviolet. $PM_{2.5}$: particulate matter, where particles have an aerodynamic diameter equal to or less than 2.5 µm (Source: WHO global air quality guidelines. Geneva: World Health Organization.) O3: ozone.

* Timing of exposure varies; method of measurement of the exposure varies.

**The provided definitions of MNH outcomes are aligned with established definitions in the literature, unless otherwise specified.

Table S4b. Reviews on air pollution and MNH outcomes (N=47).

Name of first author and year of publication	Review aim	Air pollutants	MNH Outcomes**	Number of included studies & Key Findings	Other Information
Bonzini M (2010).	To systematically synthesize the evidence on the association between maternal exposure to ambient air pollution and birth outcomes through a narrative review approach.	- Particulate matter ($PM_{2.5}$ and PM_{10})<br>-NO2<br>-CO<br>-O3	-PTB=8 studies<br>-LBW=7 studies<br>-SGA=4 studies	18 studies included in the systematic review.<br><br>-Exposure to PM_{10} during pregnancy is associated with increased risk of preterm birth (6/8 studies).<br><br>-Exposure to PM_{10} during pregnancy is associated with increased risk of low birth weight at term (2/7 studies).	Risk of bias: no evidence of quality assessment.
Bosetti C (2010).	To systematically synthesize the evidence on the association between maternal exposure to ambient air pollution and pregnancy outcomes through a narrative review approach.	- Particulate matter ($PM_{2.5}$ and PM_{10})	-PTB=13 papers<br>-LBW=17 papers<br>-SGA= 4 papers	30 studies included in the systematic review.<br><br>-Exposure to particulate matter during pregnancy is associated with increased risk of preterm birth (8/13 studies).<br><br>-Exposure to particulate matter during pregnancy is associated with increased risk of low birth weight (11/17 studies).<br><br>-Exposure to particulate matter during pregnancy is associated with increased risk of small for gestational age (2/4 studies).	Risk of bias: no evidence of quality assessment.
Veras MM (2010).	To systematically assess the evidence on the association between exposure to air pollution and reproductive, fetal and neonatal health outcomes through a narrative review approach and with a particular focus on Brazil.	- Particulate matter ($PM_{2.5}$ and PM_{10})<br>-NO<br>-SO2	-LBW=10 studies<br>-PTB=7 studies<br>-Infant mortality= 6 studies<br>-Neonatal mortality= 3 studies<br>-SGA=3 studies<br>--Other reproductive health outcomes=8 studies	44 studies included in the systematic review, of which 34 focused on human health outcomes and 10 on animal models.<br><br>-Prenatal exposure to ambient air pollution is associated with increased risk of preterm birth (6/7 studies).<br>-Prenatal exposure to ambient air pollution is associated with increased risk of low birth weight (9/10 studies).<br>-Prenatal exposure to ambient air pollution is associated with increased risk of preterm birth (5/7 studies) or reduced duration of gestation (2/7 studies).	Risk of bias: no evidence of quality assessment.

Name of first author and year of publication	Review aim	Air pollutants	MNH Outcomes**	Number of included studies & Key Findings	Other Information
				-Exposure to ambient air pollution is associated with increased risk of neonatal deaths (2/3 studies) and infant mortality (2/6 studies).	
Shah PS (2011).	To systematically review the literature on the impact of air pollution on birth outcomes among pregnancies of women exposed to various pollutants and summarize the findings through a narrative review approach.	-Sulphur dioxide (SO2).<br>-Nitric dioxide (NO2).<br>-Nitric oxide (NO).<br>-Carbon monoxide (CO).<br>-Particulate matter ($PM_{2.5}$ and PM_{10})--Ozone (O3).	-Low birth weight (LBW)= 14 studies investigate LBW in relation to SO2, 11 studies in relation to NO2, 3 studies in relation to NO, 13 studies in relation to CO, 7 studies in relation to O3, 16 studies in relation to particulate matter.<br>-Preterm birth (PTB)= 5 studies investigate PTB in relation to SO2, 6 studies in relation to NO2, 2 studies in relation to NO, 3 studies in relation to CO, 2 studies in relation to O3, 3 studies in relation to particulate matter.<br>-Small-for-gestational age (SGA)= 1 study investigates SGA in relation to SO2, 6 studies in relation to NO2, 4 studies in relation to CO, 3 studies in relation to O3, 10 studies in relation to particulate matter.	41 studies included in the systematic review.<br><br>-Exposure to SO2 during pregnancy is associated with increased risk of preterm birth (4/5 studies).<br>-Exposure to particulate matter during pregnancy is associated with SGA (4/5 studies specifically focusing on PM_{10}).<br>- Inconsistent evidence with regards to exposure to NO2 during pregnancy and low birth weight, preterm birth and SGA.<br>-Inconsistent evidence with regards to NO exposure during pregnancy and low birth weight, preterm birth and SGA.<br>-Inconsistent evidence with regards to CO exposure during pregnancy and low birth weight, preterm birth and SGA.<br>-Inconsistent evidence with regards to O3 exposure during pregnancy and low birth weight, preterm birth and SGA.	Risk of bias: Quality assessment conducted for all included studies.
Stieb DM (2012).	To systematically investigate the literature on the relationship between air pollution and birth weight and preterm birth and to perform a meta-analysis.	-NO2<br>-CO<br>-SO2<br>-O3<br>- Particulate matter ($PM_{2.5}$ and PM_{10})	Birth weight (including LBW) = 45 studies<br>Gestational age (including PTB) =19 studies<br>IUGR= 5 studies<br>SGA= 7 studies	62 studies included in the systematic review and in the meta-analysis.<br><br>- A 1 parts per billion increase of CO in entire pregnancy is associated with increased odds of low birth weight (pooled OR 1.04; 95% CI (1.02-1.06)).<br><br>- A 20 µg/m3 increase of PM_{10} in entire pregnancy is associated with increased odds of low birth weight (pooled OR 1.06; 95% CI (1.02-1.06)).<br><br>-Inconsistent evidence with regards to the association between air pollution and preterm birth.	Systematic review and meta-analysis.<br><br>Risk of bias: Quality assessment conducted for all included studies.

Name of first author and year of publication	Review aim	Air pollutants	MNH Outcomes**	Number of included studies & Key Findings	Other Information
Pedersen M (2014).	To systematically review epidemiological studies investigating associations between air pollution and hypertensive disorders of pregnancy and to perform a meta-analysis.	-NO<br>-NO2<br>-CO<br>-O3<br>-Particulate matter ($PM_{2.5}$ and PM_{10})	-Hypertensive disorders of pregnancy= 17 studies	17 studies included in the systematic review. 16 studies included in the meta-analysis.<br><br>-Pregnancy-induced hypertensive disorders were statistically significantly associated with a 5 µg/m3 increment in $PM_{2.5}$ (OR=1.47; 95% CI, 1.27, 1.68).<br><br>- Pregnancy-induced hypertensive disorders were statistically significantly associated with a 10 µg/m3 increment in NO2 (OR=1.23; 95% CI, 1.04, 1.41).<br><br>- Pregnancy-induced hypertensive disorders were statistically significantly associated with a 10 µg/m3 increment in PM_{10} (OR=1.11; 95% CI, 1.00, 1.21).	Systematic review and meta-analysis.<br><br>Risk of bias: Quality assessment conducted for all included studies.
Chen EK (2014).	To systematically synthesize the evidence on the association between exposure to ambient air pollution during pregnancy and congenital anomalies, and to perform a meta-analysis.	- Particulate matter ($PM_{2.5}$ and PM_{10})<br>-NO2<br>-SO2<br>-CO<br>-O3	-Congenital anomalies=17 studies	17 studies included in the systematic review and meta-analysis.<br><br>-The meta-analysis showed that exposure to NO2 concentrations were significantly associated with coarctation of the aorta (OR = 1.20 per 10 ppb, 95% CI, (1.02, 1.41)).	Systematic review and meta-analysis.<br><br>Risk of bias: no evidence of quality assessment.
Frutos V (2015).	To systematically synthesize the evidence on the effects of exposure to ambient air pollution on fertility outcomes in humans and mammals through a narrative review approach.	- Particulate matter ($PM_{2.5}$ and PM_{10})<br>-NO2<br>-CO<br>-O3	-Live births= 3 studies<br>-Miscarriage= 4 studies<br>-Implantation rate= 4 studies<br>-Embrio quality= 2 studies	10 studies included in the systematic review, of which 7 studies on humans and 3 experimental studies on mammals.<br><br>-Exposure to air pollution is associated with a detrimental effect on live birth rate (3/3 studies).<br><br>-Exposure to air pollution is associated with an increased risk of miscarriage (3/4 studies).	Risk of bias: Quality assessment conducted for all included studies.
Checa Vizcaino MA (2016).	To systematically synthesize the evidence on the	- Particulate matter ($PM_{2.5}$ and PM_{10})	-Live birth=3 studies<br>-Implantation rates=3 studies<br>-Miscarriage=5 studies	9 studies included in the systematic review.	Risk of bias: Quality assessment conducted for all included studies.

Name of first author and year of publication	Review aim	Air pollutants	MNH Outcomes**	Number of included studies & Key Findings	Other Information
	effects of exposure to ambient air pollution on fertility outcomes in humans through a narrative review approach.	-NO_2<br>-CO<br>-O_3		-Exposure to air pollution is associated with a detrimental effect on live birth rate (2/3 studies).<br><br>-Exposure to air pollution is associated with a detrimental effect on implantation rates (2/3 studies).<br><br>-Exposure to air pollution is associated with an increased risk of miscarriage in the general population (2 studies) and in women undergoing fertility treatments (1 study).	
Siddika N (2016).	To systematically assess the evidence on the association between prenatal exposure to air pollution and stillbirth and to perform a meta-analysis.	- Particulate matter ($PM_{2.5}$ and PM_{10})<br>-NO_2<br>-CO<br>-SO_2	-Stillbirth=13 studies	13 studies included in the systematic review and in the meta-analysis.<br><br>- The meta-analysis showed that exposure to air pollution is associated to increased risk of stillbirth as all summary effect estimates for the risk of stillbirth were systematically elevated (although not reaching statistical significance), in relation to mean prenatal exposure to NO_2 per 10 ppb (EE=1.066, 95% CI 0.965 to 1.178, n=3), CO per 0.4 ppm (EE=1.025, 95% CI 0.985 to 1.066, n=3), SO_2 per 3 ppb (EE=1.022, 95% CI 0.984 to 1.062, n=3,), $PM_{2.5}$ per 4 µg/m3 (EE=1.021, 95% CI 0.996 to 1.046, n=2) and PM_{10} per 10 µg/m3 (EE=1.014, 95% CI 0.948 to 1.085, n=2).	Systematic review and meta-analysis.<br><br>Risk of bias: Quality assessment conducted for all included studies
Hehua Z (2017).	To systematically synthesize the evidence on the association between prenatal exposure to ambient air pollution during pregnancy and incidence or prevalence of asthma from birth up to 14 years of age providing risk estimates.	- Particulate matter ($PM_{2.5}$ and PM_{10})<br>-NO_2<br>-SO_2<br>-CO<br>-O_3	-Asthma=18 studies	18 studies included in the systematic review.<br>-Prenatal exposure to air pollution is significantly associated to risk of developing asthma or wheeze in children. The overall random effects risk estimates (95% CI) of different pollutants were 1.04 (1.01–1.07) for NO_2, 1.4 (0.97–2.03) for $PM_{2.5}$ for childhood wheeze and 1.07 (1.01–1.14) for NO_2, 1 (0.97–1.03) for $PM_{2.5}$, 1.02 (0.98–1.07) SO_2 and 1.08 (1.05–1.12) for PM_{10} for childhood asthma.	Risk of bias: Quality assessment conducted for all included studies.

Name of first author and year of publication	Review aim	Air pollutants	MNH Outcomes**	Number of included studies & Key Findings	Other Information
Li X (2017).	To systematically assess the evidence on the association between prenatal exposure to air pollution (fine particulate matter) and pregnancy and/or birth outcomes, and to perform a meta-analysis.	- Particulate matter ($PM_{2.5}$)	-LBW= 15 studies<br>-PTB=14 studies	23 studies included in the systematic review and in the meta-analysis.<br><br>-The meta-analysis showed a significantly increased risk of preterm birth with interquartile range increase in $PM_{2.5}$ exposure throughout pregnancy (OR 1⁄4 1.03; 95% conditional independence (CI): 1.01, 1.05).<br><br>-The pooled OR for the association between $PM_{2.5}$ exposure, per interquartile range increment, and term low birth weight throughout pregnancy was 1.03 (95% CI: 1.02,1.03).<br><br>-The pooled ORs for the association between $PM_{2.5}$ exposure per 10 increment, and term low birth weight and preterm birth were 1.05 (95% CI: 0.98,1.12) and 1.02 (95% CI: 0.93,1.12), respectively throughout pregnancy.	Systematic review and meta-analysis.<br><br>Risk of bias: Quality assessment conducted for all included studies.
Melody SM (2018).	To synthesize the existing literature on the relationship between maternal exposure to acute short term to medium term changes in outdoor air quality (such as wildfires) and obstetric and neonatal outcomes through a narrative review approach.	-Short to medium term periods of poor air quality.	-PTB= 3 studies<br>-Birth weight= 9 studies<br>-Gestational length= 2 studies	11 studies included in the systematic review.<br><br>-Inconsistent evidence with regards to exposure to acute changes in air quality (air pollution exacerbation events) during pregnancy and birth weight/low birth weight.<br><br>-Exposure to acute changes in air quality during pregnancy is associated with increased risk of preterm birth (3/3 studies).	Risk of bias: Quality assessment conducted for all included studies.
Nicoll R (2018).	To systematically assess the evidence on the association between prenatal	- Particulate matter (PM_{10})<br>-NO2<br>-SO2	Congenital heart disease=4 studies	69 studies included in the systematic review of which 4 studies focusing on ambient air pollution.	Risk of bias: no evidence of quality assessment.

Name of first author and year of publication	Review aim	Air pollutants	MNH Outcomes**	Number of included studies & Key Findings	Other Information
	exposure to environmental contaminants including ambient air pollution and congenital heart disease through a narrative review approach.			-Prenatal exposure to NO2 is associated with increased risk of congenital heart disease (2/4 studies).<br><br>-Prenatal exposure to SO2 is associated with increased risk of congenital heart disease (1/4 studies).<br><br>-Prenatal exposure to PM_{10} is associated with increased risk of congenital heart disease, particularly atrial septal defects and patent ductus arteriosus (2/4 studies).	
Klepac P (2018).	To systematically assess the evidence on the association between prenatal exposure to ambient air pollution and pregnancy outcomes, and to perform a meta-analysis.	- Particulate matter ($PM_{2.5}$ and PM_{10})<br>-NO2<br>-CO<br>-SO2<br>-O3	-PTB*<br>-LBW*<br>-SGA*<br><br>**number of studies per outcome unclear*	96 studies included in the systematic review and 28 studies included in the meta-analysis.<br><br>-The meta-analysis showed that exposure to particulate matter and ozone over the entire pregnancy was significantly associated with higher risk for preterm birth: the pooled effect estimates were 1.09 (1.03- 1.16) per 10 µg/m3 increase in PM_{10}, 1.24 (1.08-1.41) per 10 µg/m3 increase in $PM_{2.5}$ and 1.03 (1.01-1.04) per 10 ppb increase in O3.	Systematic review and meta-analysis.<br><br>Risk of bias: Quality assessment conducted for all included studies
Porpora MG (2019).	To systematically identify and summarize the published studies on the possible role of environmental toxicant exposure in preterm birth through a narrative review approach.	-NO2<br>-NO<br>-CO<br>-SO2<br>-O3<br>- Particulate matter ($PM_{2.5}$ and PM_{10})<br>-Drinking water contaminants<br>-Persistent and non-persistent organic compounds<br>-Tobacco smoke<br>-Toxic metals	-PTB= 78 studies	78 studies included in the systematic review.<br><br>-Exposure to air pollutants during pregnancy (CO, NO, NO2, SO2, O3) is associated with increased risk of preterm birth (5/5 studies).<br><br>-Exposure to particulate matter (PM_{10}-$PM_{2.5}$) is associated with increased risk of preterm birth (2/2 studies).<br><br>-Inconsistent evidence with regards to exposure to drinking water contaminants and preterm birth.<br>-Inconsistent evidence with regards to exposure to organic compounds and preterm birth.	Risk of bias: no evidence of quality assessment.

Name of first author and year of publication	Review aim	Air pollutants	MNH Outcomes**	Number of included studies & Key Findings	Other Information
				-Exposure to tobacco smoke during pregnancy is associated with increased risk of preterm birth as well as fetal growth restriction (20/20 studies).<br><br>-Exposure to toxic metals during pregnancy is associated with increased risk of preterm birth (2/2 studies).	
Bekkar B (2020).	To systematically assess the association between exposure to heat and air pollution to birth outcomes in the United States using Arksey and O'Malley's methodological framework for scoping reviews.	-O3<br>-Particulate matter ($PM_{2.5}$)<br>-Temperature (heat).	-PTB= 29 studies (24 studies investigate PTB in relation to air pollution, 5 studies in relation to heat)<br>-LBW=32 studies (29 studies investigate LBW in relation to air pollution, 3 studies in relation to heat)<br>-Stillbirth= 7 studies (5 studies investigate stillbirth in relation to air pollution, 2 studies in relation to heat)<br><br>*Note: articles analysing non-US populations were excluded.*	68 studies included in the systematic review.<br><br>-Exposure to air pollutants during pregnancy is associated with increased risk of preterm birth (19/24 studies; 7.3 million total births; increased risk median 11.5% range (2.0-19.0) for whole pregnancy PM2.5 exposure).<br><br>-Exposure to air pollutants during pregnancy is associated with increased risk of low birth weight (25/29 studies; 18.5 million total births; increased risk median 10.8% range (2.0-3.6) for whole pregnancy PM 2.5 exposure).<br><br>-Exposure to air pollutants during pregnancy is associated with increased risk of stillbirth (4/5 studies; 5.1 million total births; increased risk median 14.5% range (6.0-23.0) for whole pregnancy PM2.5 exposure)	Scoping review.<br><br>Risk of bias: no evidence of quality assessment.
Zhang H (2020).	To systematically review the literature on the effect of exposure to air pollution on the risk of gestational diabetes and to perform a meta-analysis.	-NO2<br>-SO2<br>-O3<br>-Particulate matter ($PM_{2.5}$ and PM_{10})	-Gestational diabetes (GD)= 13 studies	13 studies included in the systematic review and in the meta-analysis.<br><br>-A 10 µg/m3 increase in first trimester SO2 (standardized OR = 1.392, 95% CI: 1.010, 1.773) is associated with gestational diabetes.<br><br>-No significant association was found in the first or second trimester exposure to O3. Pre-pregnancy exposure to O3 is inversely associated with GDM risk (standardized OR = 0.981, 95% CI: 0.977, 0.985).	Systematic review and meta-analysis.<br><br>Risk of bias: Quality assessment conducted for all included studies.

Name of first author and year of publication	Review aim	Air pollutants	MNH Outcomes**	Number of included studies & Key Findings	Other Information
				-No significant association was observed in pre-pregnancy, first trimester or second trimester exposure to $PM_{2.5}$ or PM_{10} or NO2.	
Bai W (2020).	To systematically investigate the effect of trimester-specific exposure to air pollution on hypertensive disorders of pregnancy and gestational diabetes and to perform a meta-analysis.	-NO2<br>-NO<br>-CO<br>-SO2<br>-O3<br>-Particulate matter ($PM_{2.5}$ and PM_{10})	-Hypertensive disorders of pregnancy=23 studies<br>-GD= 12 studies	33 studies included in the systematic review and in the meta-analysis.<br><br>-Exposure during the first trimester to PM_{10}, is significantly associated with increased risk of hypertensive disorders of pregnancy (RR = 1.07, 95% CI: 1.02–1.12 per 10 μg/m3).<br><br>-Exposure during the first trimester to SO2 is significantly associated with increased risk of GD (RR = 1.04, 95% CI: 1.00–1.08 per 1 parts per billion increment).<br><br>-Exposure during the first trimester to $PM_{2.5}$ is significantly associated with increased risk of hypertensive disorders of pregnancy, specifically pre-eclampsia (RR = 0.97, 95% CI: 0.95–1.00 per 5 μg/m3).<br><br>-Exposure to $PM_{2.5}$ during the entire pregnancy is significantly associated with increased risk of hypertensive disorders of pregnancy (RR = 1.18, 95% CI: 1.02–1.34 per 5 μg/m3).	Systematic review and meta-analysis.<br>Risk of bias: Quality assessment conducted for all included studies.

Name of first author and year of publication	Review aim	Air pollutants	MNH Outcomes**	Number of included studies & Key Findings	Other Information
Simoncic V (2020).	To systematically investigate the literature on the relationship between air pollution and adverse birth outcomes in Europe and to perform a meta-analysis.	-NO2<br>-Particulate matter ($PM_{2.5}$ and PM_{10})	-LBW= 7 studies<br>-PTB=13 studies<br>-SGA=5 studies	14 studies included in the systematic review. 10 studies included in the meta-analysis.<br><br>-The findings of the systematic review do not show statistically significant results, although a trend is apparent in that NO2 exposure during the whole pregnancy seems to increase the prevalence of LBW.<br><br>-The findings of the meta-analysis do not reveal statistically significant results.	Systematic review and meta-analysis.<br><br>Risk of bias: no evidence of quality assessment.
Carvalho MA (2020).	To systematically investigate the association between air pollution and head size at birth through a narrative review approach.	-PAH (polycyclic aromatic hydrocarbons)<br>-NO2<br>-NO<br>-CO<br>- Particulate matter ($PM_{2.5}$ and PM_{10})	-Impact on head size at birth	13 studies included in the systematic review.<br><br>-Inconsistent evidence on the relationship between prenatal exposure to air pollution and head size at birth.	Observational study and systematic review.<br>Risk of bias: no evidence of quality assessment.
Padula AM (2020).	To systematically assess the evidence on the association between prenatal exposure to air pollution and heavy metals and infant and child health outcomes through a narrative review approach.	- Particulate matter ($PM_{2.5}$ and PM_{10})<br>-NO2<br>-SO2<br>-CO<br>-O3	-LBW=4 studies<br>-SGA= 2 studies<br>-PTB=3 studies	30 studies included in the systematic review.<br><br>-The prenatal exposure to air pollutants is associated with LBW (3/4 studies) and PTB (3/3 studies), while the evidence for SGA is inconclusive.<br><br>-When combining the effects of air pollution and socio-economic status on birth outcomes, the associations appear to be stronger.	Risk of bias: no evidence of quality assessment.
Tang X (2020).	To systematically assess the evidence on the association between exposure to air pollution and	- Particulate matter ($PM_{2.5}$ and PM_{10})<br>-NO2<br>-NOx	-GD= 13 studies	13 studies included in the systematic review and in the meta-analysis.<br><br>-The meta-analysis showed that exposure to $PM_{2.5}$ was not associated with the increased risk of GDM (adjusted	Systematic review and meta-analysis.

Name of first author and year of publication	Review aim	Air pollutants	MNH Outcomes**	Number of included studies & Key Findings	Other Information
	gestational diabetes and to perform a meta-analysis.	-SO2<br>-CO<br>-O3		OR 1.03, 95% CI 0.99 to 1.06) but the subgroup analysis showed positive correlation of $PM_{2.5}$ exposure in the second trimester with an increased risk of GDM (combined OR 1.07, 95% CI 1.00 to 1.13).<br><br>-Among other pollutants, significant associations between GDM and NO2 (OR 1.05, 95% CI 1.01 to 1.10), NOx(OR 1.03, 95% CI 1.01 to 1.05), and SO2 (OR 1.09, 95% CI 1.03 to 1.15) were noted.	Risk of bias: Quality assessment conducted for all included studies.
Steinle S (2020).	To assess the evidence on the association between prenatal exposure to particulate matter and birth weight or changes in birth weight, through a narrative review approach.	- Particulate matter ($PM_{2.5}$ and PM_{10})	-LBW=9 studies<br>-Changes in birth weight= 4 studies	13 studies included in the systematic review.<br><br>- Exposure to particulate matter pollution during pregnancy is associated with low birth weight, with most meta-studies finding statistically significant point estimates (6/9 studies).<br><br>-Exposure to particulate matter pollution during pregnancy is associated with reductions in birth weight (4/4 studies).	Risk of bias: no evidence of quality assessment.
Zhang H (2021).	To perform a systematic review and meta-analysis of the association between air pollution and stillbirth.	-SO2<br>-NO2<br>-CO<br>-O3<br>-Particulate matter ($PM_{2.5}$ and PM_{10})	-Stillbirth= 15 studies	15 studies included in the systematic review.<br><br>-A 10 mg/m3 increase of $PM_{2.5}$ in the third trimester and entire pregnancy were associated with increased odds of stillbirth, with the standardized ORs and 95% CIs being 1.094 (1.008-1.180) and 1.103 (1.074-1.131), respectively.<br><br>-Exposure to CO during the third trimester and exposure to O3 during the first trimester also increased the odds of stillbirth, with the standardized ORs and 95% CIs of 1.0009 (1.0001-1.0017) and 1.028 (1.001-1.055), respectively.	Systematic review and meta-analysis.<br>Risk of bias: Quality assessment conducted for all included studies.

Name of first author and year of publication	Review aim	Air pollutants	MNH Outcomes**	Number of included studies & Key Findings	Other Information
				-First trimester O3 exposure was significantly associated with stillbirth, with the standardized OR and 95% CI were 1.002 and 1.001-1.004, respectively.	
Aghababaeian H (2021).	To systematically investigate the literature on the impacts of dust storms on human health through a narrative review approach.	- Particulate matter ($PM_{2.5}$ and PM_{10})	-PTB=1 study<br>-Birth weight (including LBW) = 2 studies	140 studies included in the systematic review. 3 studies provided estimates for MNH outcomes.<br><br>-Exposure to dust storms during pregnancy is associated to increased risk of preterm birth (1 study) and to increased risk of LBW (1 study).	Risk of bias: Quality assessment conducted for all included studies.
Amjad S (2021).	To systematically summarize the evidence on the association between maternal exposure to wildfires and adverse pregnancy outcomes through a narrative review approach.	- Particulate matter ($PM_{2.5}$ and PM_{10})	-Birth weight=7 studies<br>-Gestational length=4 studies<br>-SGA= 2 studies<br>-Infant mortality= 1 study	8 studies included in the systematic review.<br><br>-Exposure to wildfire smoke during pregnancy is associated with a reduction in mean birth weight (range –4.8 grams per 1 µg/m3 increase in gestational average daily smoke exposure to 18.0 grams for overall exposure) (6/7 studies).<br><br>-Inconsistent evidence with regards to the association between wildfire smoke exposure and SGA.<br><br>-Inconsistent evidence with regards to the association between wildfire smoke exposure and PTB.	Risk of bias: Quality assessment conducted for all included studies.
Henry S (2021).	To systematically synthesize the evidence on the risk of respiratory related healthcare visits among children aged 0-18 years following exposure to wildfire smoke.	- Particulate matter ($PM_{2.5}$ and PM_{10})	-Effect on respiratory symptoms children 0-18 years old.	16 studies included in the systematic review.<br><br>-Exposure to wildfire smoke is associated to increased respiratory visits to the emergency department (8/16 studies).	Risk of bias: Quality assessment conducted for all included studies.<br><br>No specific mentions of newborns (0-28 days).
Cao L (2021).	To systematically synthesize the evidence on the	- Particulate matter ($PM_{2.5}$ and PM_{10})	-Hypertensive disorders of pregnancy (gestational	17 studies included in the systematic review and meta-analysis.	Systematic review and meta-analysis.

Name of first author and year of publication	Review aim	Air pollutants	MNH Outcomes**	Number of included studies & Key Findings	Other Information
	association between maternal exposure to particulate matter and hypertensive disorders of, and to perform a meta-analysis.		hypertension, pre-eclampsia) =17 studies	- The overall meta-analysis showed that the risk of hypertensive disorders of pregnancy is significantly associated with per 5 mg/m3 increase in $PM_{2.5}$ exposure during the first trimester and PM_{10} exposure throughout pregnancy, with odds ratios 1.06 (95% confidence interval [CI]: 1.01,1.12) and 1.04 (95% CI: 1.02, 1.07), respectively.<br><br>-Exposure to $PM_{2.5}$ exposure during the first and second trimester and to PM_{10} exposure during the first trimester increased the incidence of hypertensive disorders of pregnancy; the summary ORs were 1.11 (95% CI: 1.01,1.23), 1.16 (95% CI: 1.05,1.29), and 1.04 (95% CI: 1.02 ,1.07), respectively.	Risk of bias: Quality assessment conducted for all included studies.
Ma Z (2021).	To systematically synthesize the evidence on the association between prenatal exposure to ambient air pollution and congenital heart diseases, and to perform a meta-analysis.	- Particulate matter ($PM_{2.5}$ and PM_{10})<br>-NO2<br>-SO2<br>-CO<br>-O3	-Congenital heart disease= 24 studies	24 studies included in the systematic review and in the meta-analysis.<br><br>-Exposure to air pollutants is positively associated to development of ventricular septal defects, with an OR (95%CI) for PM_{10}, $PM_{2.5}$, and O3 of 1.057(1.005–1.109), 1.208(1.080–1.337), and 1.205(1.101–1.310), respectively.	Systematic review and meta-analysis.<br><br>Risk of bias: Quality assessment conducted for all included studies.
Uwak I (2021).	To systematically synthesize the evidence on the association between prenatal exposure to particulate matter air pollution and birth weight, and to perform a meta-analysis.	-Particulate matter ($PM_{2.5}$ and PM_{10})	-Birth weight=53 studies	18 studies included in the systematic review and 33 studies included in the meta-analysis.<br><br>-The meta-analysis showed that for every 10 µg/m3 increase in PM_{10} exposure in the 3rd trimester or the entire pregnancy, there is a 6.57 g decrease (I^2: 0%, 95% CI: − 10.66, − 2.48) or 8.65 g decrease in birth weight (I^2: 84%, 95% CI: − 16.83, − 0.48), respectively.	Systematic review and meta-analysis.<br><br>Risk of bias: Quality assessment conducted for all included studies.
Isaevska E (2021).	To systematically assess the evidence on the association	- Particulate matter ($PM_{2.5}$ and PM_{10})	-DNA methylation status in cord blood/placenta=30 studies	32 studies included in the systematic review.	Risk of bias: no evidence of quality assessment.

Name of first author and year of publication	Review aim	Air pollutants	MNH Outcomes**	Number of included studies & Key Findings	Other Information
	between exposure to air pollutants during the first 1000 days of life (from conception to 2 years) and changes in DNA methylation pattern in cord or newborn blood and/or in children through a narrative review approach.	-NO_2<br>-SO_2<br>-CO<br>-O_3	-DNA methylation studies in childhood= 5 studies	-Exposure to ambient air pollution exposure during pregnancy (particularly in the first trimester) is associated with global loss of methylation in newborn cord blood and placenta, indicating the beginning of the pregnancy as a potential period of susceptibility.	
Xie G (2021).	To systematically assess the evidence on the association between prenatal exposure to air pollution (fine particulate matter) and stillbirth and to perform a meta-analysis.	- Particulate matter ($PM_{2.5}$)	-Stillbirth=7 studies	7 studies included in the systematic review and 6 studies included in the meta-analysis.<br><br>-The meta-analysis showed a positive association between the maternal exposure to $PM_{2.5}$ (per 10 µg/m3 increased) in the entire pregnancy (OR: 1.15, 95% CI: 1.07–1.25) and third trimester (OR: 1.09, 95% CI: 1.01–1.18) and stillbirth.	Systematic review and meta-analysis.<br><br>Risk of bias: Quality assessment conducted for all included studies.
Yu G (2021).	To systematically assess the evidence on the association between prenatal exposure to air pollution (particulate matter) and congenital anomalies, and to perform a meta-analysis.	- Particulate matter ($PM_{2.5}$ and PM_{10})	-Congenital anomalies= 30 studies	30 studies included in the systematic review and meta-analysis.<br><br>-The meta-analysis showed that prenatal exposure to PM_{10} is associated with an increased risk of congenital heart disease, neural tube defects, and cleft lip with or without cleft palate (OR per 10 µg/m3 = 1.05, 95% CI, 1.03–1.07; OR per 10 µg/m3 = 1.04, 95% CI, 1.01–1.06; OR per 10 µg/m3 = 1.03, 95% CI, 1.01–1.06).	Systematic review and meta-analysis.<br><br>Risk of bias: Quality assessment conducted for all included studies
Ravindra K (2021).	To systematically assess the evidence on the association between prenatal	- Particulate matter ($PM_{2.5}$ and PM_{10})<br>-NO_2	-Congenital anomalies= 26 studies	26 studies included in the systematic review and in the meta-analysis.<br>-The meta-analysis showed that prenatal exposure to air pollution is associated with increased risk of	Systematic review and meta-analysis.

Name of first author and year of publication	Review aim	Air pollutants	MNH Outcomes**	Number of included studies & Key Findings	Other Information
	exposure to air pollution and congenital anomalies, and to perform a meta-analysis.	-CO<br>-SO2<br>-O3		congenital anomalies. Exposure to NO2 and $PM_{2.5}$ is associated with the risk of pulmonary valve stenosis with OR=1.74 (95%CI: 1.68–1.81) and OR=1.42 (95%CI: 1.36–1.48) respectively. The risk of developing tetralogy of Fallot (TOF) was observed to be associated with PM2.5 with OR=1.52 (95%CI: 1.44–1.60). Exposure to SO2 is associated with risk of orofacial defects OR=1.55 (95%CI=1.47-1.63).	Risk of bias: Quality assessment conducted for all included studies
Volk HE (2021).	To systematically assess the evidence on the association between prenatal exposure to air pollution and neurodevelopmental outcomes.	- Particulate matter ($PM_{2.5}$ and PM_{10})<br>-NO2<br>-CO<br>-SO2<br>-O3	-Cognitive abilities=38 studies<br>-Attention deficit and hyperactivity disorder=11 studies<br>-Autism spectrum disorder=18 studies<br>-Mood disorders=12 studies<br>-Brain abnormalities by neuroimaging= 8 studies	68 studies included in the systematic review.<br><br>-Inconclusive results with regards to the association between prenatal exposure to air pollution and cognitive abilities, development of attention deficit and hyperactivity disorder, mood disorders and brain abnormalities.	Risk of bias: no evidence of quality assessment.
Ghosh R (2021).	To systematically assess the evidence on the association between exposure to air pollution particulate matter and perinatal outcomes, and to perform a meta-regression.	- Particulate matter ($PM_{2.5}$)	-Birth weight=44 studies<br>-LBW= 40 studies<br>-PTB= 40 studies	124 studies included in the systematic review and in the meta-analysis.<br><br>-The meta-regression showed pooled estimates indicating 22 grams (95% UI: 12, 32) lower birth weight, 11% greater risk of LBW (1.11, 95% UI: 1.07, 1.16), and 12% greater risk of PTB (1.12, 95% UI: 1.06, 1.19), per 10 µg/m3 increment in ambient $PM_{2.5}$.<br><br>-Globally, an estimated 15.6% (95% UI: 15.6, 15.7) of all LBWs and 35.7% (95% UI: 35.6, 35.9) of all preterm births were attributable to total $PM_{2.5}$, equivalent to 2,761,720 (95% UI: 2,746,713 to 2,776,722) and 5,870,103 (95% UI: 5,848,046 to 5,892,166) newborns in 2019, respectively.	Systematic review and meta-analysis.<br><br>Risk of bias: Quality assessment conducted for all included studies
Markozannes G (2022).	To perform an umbrella review to evaluate the strength of the association between exposure to ambient air pollution	- Particulate matter ($PM_{2.5}$ and PM_{10})<br>-NO2<br>-SO2<br>-CO<br>-O3	- Hypertensive disorders of pregnancy (gestational hypertension, pre-eclampsia) =1 study<br>-SGA=3 studies<br>-LBW=3 studies	75 studies included in the umbrella review; 4 studies focused on MNH outcomes.<br><br>- 10 µg/m increase of $PM_{2.5}$ levels was associated with an increased risk of having an infant born small for the gestational age, (long-term exposure-entire pregnancy:	Umbrella review.<br><br>Risk of bias: Quality assessment conducted for all included studies.

Name of first author and year of publication	Review aim	Air pollutants	MNH Outcomes**	Number of included studies & Key Findings	Other Information
	and adverse health outcomes.			OR: 1.151, 95% CI: 1.104 to 1.200; long-term exposure-first trimester: OR: 1.074, 95% CI: 1.046 to 1.103; long-term exposure-last trimester: OR: 1.062, 95% CI: 1.042 to 1.083).<br><br>- During the entire pregnancy, an increased risk for low birth weight was associated with a 5-ppb increase of SO2 levels (24 h average; OR: 1.035, 95% CI: 1.021 to 1.049), whereas a 10 µg/m increase of $PM_{2.5}$ levels during the third trimester (long-term exposure) was associated with an increased risk for hypertension in pregnancy (OR: 2.177, 95% CI, 1.710 to 2.773).	
Lin LZ (2022).	To systematically synthesize the evidence on the association between prenatal exposure to particulate matter, as well as exposure during the first and second year after birth and development of autism spectrum disorders, and to perform a meta-analysis.	- Particulate matter ($PM_{2.5}$ and PM_{10})	-Autism spectrum disorders= 31 studies	31 studies included in the systematic review; 20 studies included in the meta-analysis.<br><br>-Exposure to particulate matter of 2.5 µm or less (PM2.5) during the prenatal period (OR, 1.32 [95%CI, 1.03–1.69]), the first year after birth (OR, 1.62 [95%CI, 1.22–2.15]) and the second year after birth (OR, 3.13 [95%CI, 1.47–6.67]) is associated with increased risk of autism spectrum disorders.	Systematic review and meta-analysis.<br><br>Risk of bias: Quality assessment conducted for all included studies.
Gong C (2022).	To systematically synthesize the evidence on the effects of prenatal exposure to particulate matter on changes in grams of term birth weight, and to perform a meta-analysis.	- Particulate matter ($PM_{2.5}$)	-Changes in grams of birth weight at term =62 studies	62 studies included in the systematic review and 31 studies included in the meta-analysis.<br><br>-The meta-analysis showed that birth weight at term is negatively associated with $PM_{2.5}$ exposure (per 10 µg/m3 increment) during the entire pregnancy ($\beta = -16.54$ g), but with high heterogeneity ($I^2 = 95.6\%$).	Systematic review and meta-analysis.<br><br>Risk of bias: Quality assessment conducted for all included studies.

Name of first author and year of publication	Review aim	Air pollutants	MNH Outcomes**	Number of included studies & Key Findings	Other Information
Nyadanu DN (2022).	To perform an umbrella review to evaluate the strength of the association between exposure to ambient air pollution and adverse birth outcomes.	- Particulate matter ($PM_{2.5}$ and PM_{10})<br>-NO2<br>-SO2<br>-CO<br>-O3	- LBW and changes in birth weight at term= 26 studies<br>-PTB= 21 studies<br>-Stillbirth=9 studies<br>-Miscarriage=2 studies	23 studies included in the umbrella review.<br><br>-The prenatal exposure to $PM_{2.5}$ showed more consistent positive associations to the outcomes of interest compared to other pollutants.<br><br>-For whole pregnancy exposure, a more consistent positive association was found for $PM_{2.5}$ and birth weight reductions, particulate matter and miscarriage, and SO2 and LBW.<br><br>-Other exposure-outcome associations mostly showed less consistent positive associations and few unclear directions of associations. Almost all associations showed probable evidence.	Umbrella review.<br><br>Risk of bias: Quality assessment conducted for all included studies.
Zhu W (2022).	To systematically assess the evidence on the association between exposure to ambient air pollution (particulate matter) and miscarriage, and to perform a meta-analysis.	- Particulate matter ($PM_{2.5}$ and PM_{10})	-Miscarriage=6 studies	6 studies included in the systematic review and in the meta-analysis.<br><br>-The meta-analysis showed that exposure to particulate matter is associated with increased risk of miscarriage. The pooled relative risks for miscarriage risk associated with a 10 µg/ m3 increase in $PM_{2.5}$ and in PM_{10} were 1.20 (95%CI: 1.01–1.40) and 1.09 (95%CI: 1.02–1.15), respectively.	Systematic review and meta-analysis.<br><br>Risk of bias: Quality assessment conducted for all included studies
Yue D (2022).	To systematically assess the evidence on the association between prenatal exposure to air pollution and development of childhood eczema,	- Particulate matter ($PM_{2.5}$ and PM_{10})<br>-NO2<br>-SO2	-Childhood eczema= 12 studies	12 studies included in the systematic review and in the meta-analysis.<br><br>-The meta-analysis showed that prenatal exposure to NO2 (per 10 µg/m3 increased) is significantly associated to development of childhood eczema, with a pooled risk estimate of 1.13 (95% CI: 1.06-1.19), but no association was observed between exposure to PM_{10}, $PM_{2.5}$, and SO2 and the risk of eczema in offspring.	Systematic review and meta-analysis.<br><br>Risk of bias: Quality assessment conducted for all included studies

Name of first author and year of publication	Review aim	Air pollutants	MNH Outcomes**	Number of included studies & Key Findings	Other Information
	and to perform a meta-analysis.				
Boyd R (2022).	To systematically assess the evidence on the association between prenatal environmental exposures including exposure to air pollution and development of congenital heart disease through a narrative review approach.	- Particulate matter ($PM_{2.5}$ and PM_{10})<br>-NO<br>-SO2	-Congenital heart disease=5 studies	46 studies included in the systematic review, of which 5 focusing on ambient air pollution.<br><br>-Prenatal exposure to CO, NO and SO2 is associated with increased risk of congenital heart disease (2 studies).<br><br>-Prenatal exposure to $PM_{2.5}$ is associated with increased risk of congenital heart disease (1 study).	Risk of bias: no evidence of quality assessment.
Yu Z (2022).	To systematically assess the evidence on the association between prenatal exposure to particulate matter and preterm birth, and to perform a meta-analysis.	- Particulate matter ($PM_{2.5}$ and PM_{10})	-PTB= 84 studies	84 studies included in the systematic review and in the meta-analysis.<br><br>- The meta-analysis for long-term effect of prenatal exposure to particulate matter showed that per 10 µg/m3 increase in $PM_{2.5}$ and PM_{10} during the entire pregnancy the pooled odds ratios were 1.084 (95% CI: 1.055–1.113) and 1.034 (95% CI: 1.018–1.049).<br><br>-The meta-analysis for short-term effect of prenatal exposure to particulate matter showed that with a 10 µg/m3 increment in $PM_{2.5}$ on lag day 2 and 3, the pooled ORs and 95% CIs were 1.003 (1.001–1.004) and 1.003 (1.001–1.005), with I^2 of 65.30% and 76.60%. For PM_{10} the pooled OR was 1.001 (95% CI: 1.000, 1.001).	Systematic review and meta-analysis.<br><br>Risk of bias: Quality assessment conducted for all included studies
Evans J (2022).	To systematically assess what is known about birth outcomes, health, and health care needs of childbearing women during and	-Ambient air pollution from wildfires (without specification of individual pollutants)	-Birth weight= 8 studies<br>-PTB= 8 studies<br>-Maternal access to health care= 4 studies<br>-Congenital anomalies= 2 studies<br>-Secondary sex ratio= 2 studies<br>-Gestational diabetes= 3 studies	16 studies included in the systematic review.<br><br>- Prenatal exposure to wildfires is associated with a decrease in birth weight (6/8 studies).<br><br>-Inconclusive results with regards to the association between prenatal exposure to wildfires and preterm	Integrative review.<br><br>Risk of bias: Quality assessment conducted for all included studies

Name of first author and year of publication	Review aim	Air pollutants	MNH Outcomes**	Number of included studies & Key Findings	Other Information
	after exposure to wildfire through an integrative review approach.		-Mental health impacts= 3 studies<br>-Breastfeeding=1 study	birth, congenital anomalies, secondary sex ratio, gestational diabetes.<br><br>-Exposure to wildfires did not negatively impacts access to health care services (4/4 studies) although in one study the quality of counselling on the fires received was not considered adequate.<br><br>-Exposure to wildfires during pregnancy is associated with symptoms of post-traumatic stress disorders, and feelings of trauma and despair (3/3 studies).<br><br>-Exposure to wildfires postpartum is associated with reduced access to lactation support and lack of safe and private places, with declining breastfeeding rates during and after evacuation (1 study).	
Blanc N (2023).	To systematically synthesize the evidence on the effects of preconception ambient air pollution exposure on children's health outcomes, and to perform a meta-analysis.	- Particulate matter ($PM_{2.5}$ and PM_{10})<br>-SO2<br>-NO2<br>-CO<br>-O3	-IUGR*<br>-Congenital anomalies*<br>-PTB*<br>-SGA*<br>-NICU admissions*<br>-Perinatal mortality*<br>-LBW*<br>-Childhood pneumonia*<br>-Autism spectrum disorder*<br>-Paediatric skin disease*<br><br>**number of studies per outcome unclear*	22 studies included in the systematic review.<br><br>-Exposure to $PM_{2.5}$, PM_{10} , O3 is associated with IUGR.<br><br>-Preconception exposure to $PM_{2.5}$, PM_{10} , O3 and SO2 is associated to increased risk of preterm birth, low birth weight and diagnosis of autism spectrum disorder.<br><br>-The meta-analysis that during 3-month preconception period, a 10 µg/m3 increase in PM_{10} and $PM_{2.5}$ was associated with relative risk of birth defects of 1.06 (95% confidence interval (CI): 1.00, 1.02) and 1.14 (95% CI: 0.82, 1.59), respectively.<br><br>-Unclear evidence with regards to the association between perinatal mortality and preconception exposure to air pollution.	Systematic reviews and meta-analysis.<br><br>Risk of bias: Quality assessment conducted for all included studies.

MNH: maternal and newborn health. LBW: low birth weight. PTB: preterm birth. SGA: small for gestational age. GD: gestational diabetes. IUGR: intrauterine growth restriction. SO2: sulphur dioxide. $PM_{2.5}$: particulate matter, where particles have an aerodynamic diameter equal to or less than 2.5 µm (Source: WHO global air quality guidelines. Geneva: World Health Organization.). PM_{10}: particulate matter, where particles have an aerodynamic diameter equal to or less than 10 µm (Source: WHO global air quality guidelines. Geneva: World Health Organization). CO: carbon monoxide. O3: ozone. NO: nitric oxide. NO2: nitric dioxide. PAH: polycyclic aromatic hydrocarbons.

* Timing of exposure varies; method of measurement of the exposure varies.

**The provided definitions of MNH outcomes are aligned with established definitions in the literature, unless otherwise specified.

Table S4c. Reviews on disasters and MNH (N=7).

Name of first author and year of publication	Review aim	Disaster type	MNH Outcomes**	Number of included studies & Key Findings	Other Information
Harville E (2010).	To systematically examine the literature on the effects of disasters on perinatal health through a narrative review approach.	-Hurricanes<br>-Floods	PTB= 4 studies (2 studies with hurricane as the exposure, 2 studies with flood as the exposure)<br>-Birth weight (including LBW) = (2 studies with hurricane as the exposure, 2 studies with flood as the exposure)<br>-SGA= 1 study (with flood as the exposure)<br>-Congenital anomalies= 2 studies (1 study with hurricane as the exposure and 1 study with flood as the exposure)<br>-Maternal mental health= 3 studies (with hurricane as the exposure)<br>-Miscarriage=2 studies (with flood as the exposure)<br><br>*Note: no definitions provided for MNH outcomes.*	45 studies included in the systematic review, of which 7 focusing on hurricanes and 3 on floods.<br><br>-Exposure to hurricanes during pregnancy is not associated with increased risk of preterm birth (2/2 studies); inconclusive results with regards to the association between exposure to hurricanes during pregnancy and reduction in birth weight; inconclusive results with regards to the association between exposure to hurricanes and congenital anomalies; exposure to hurricanes during pregnancy is associated with increased risk of PTSD among mothers (3 studies) that increased with increased exposure to the event.<br><br>-Inconclusive results with regards to the association between exposure to floods during pregnancy and preterm births; exposure to floods during pregnancy is associated with increased risk of reduction of birth weight (2/2 studies); inconclusive results with regards to the association between exposure to floods and SGA; exposure to floods during pregnancy is not associated with increased risk of congenital anomalies; exposure to floods during pregnancy is associated with increased risk of miscarriage (2/2 studies).	Risk of bias: no evidence of quality assessment.
Zotti ME (2013).	To systematically assess the literature on US-disaster related reproductive health outcomes through a narrative review approach.	- Hurricanes<br>-Floods	-Fetal distress= 1 study (with hurricane as the exposure)<br>-PTB=4 studies (3 studies with hurricane as the exposure and 1 study with flood as the exposure)<br>-LBW=4 studies (3 studies with hurricane as the exposure and 1 study with flood as the exposure)<br>-Maternal mental health=1 study (with hurricane as the exposure)<br><br>*Note: no definitions provided for MNH outcomes.*	15 studies included in the systematic review, of which 4 focusing on hurricanes and 1 on floods.<br><br>-Exposure to hurricanes during pregnancy is associated with an increased risk of preterm birth (2/3 studies), of a reduction in birth weight (2/3 studies) and of fetal distress (1 study) as well as of maternal mental health issues (1 study).<br><br>-Only 1 study investigated flood as the exposure during pregnancy finding an associated between the exposure and increased risk of preterm birth and of a reduction in birth weight.	Risk of bias: no evidence of quality assessment.

Name of first author and year of publication	Review aim	Disaster type	MNH Outcomes**	Number of included studies & Key Findings	Other Information
Saulnier DD (2015).	To systematically assess the literature on the health effects of prenatal exposure to disasters through a narrative review approach.	-Hurricanes<br>-Floods	-Short- and long-term health effects following prenatal exposure to disasters	47 studies included in the systematic review, of which 3 focusing on hurricanes and 1 on floods.<br><br>-Prenatal exposure to hurricanes is associated with increased risk of fetal distress (1 study), of autism (1 study), and of diabetes and hypertension later in life (1 study).<br><br>-Only 1 study investigated prenatal exposure to floods in relation to schizophrenia finding no evidence of an association.	Risk of bias: no evidence of quality assessment.
Mallet LH (2018).	To systematically present the findings on flood related risks during pregnancy and childhood through a narrative review approach.	-Floods	Multiple outcomes, including:<br>-Maternal mortality=2 studies<br>-Perinatal mortality= 2 studies<br>-Under 5 mortality= 1 study<br>-Fetal distress= 1 study<br>-LBW= 3 studies<br>-Miscarriage=2 studies<br>-Maternal mental health= 4 studies<br><br>*Note: no definitions provided for MNH outcomes.*	141 studies included in the systematic review.<br><br>-Exposure to flooding during pregnancy is associated with increased risk of mortality among pregnant women (2 studies).<br><br>-Exposure to flood is associated with increased risk of perinatal mortality (2 studies).<br><br>-Exposure to flooding is associated with increased risk of under 5 mortality (2 studies).<br><br>-Exposure to floods during pregnancy is associated with increased risk of fetal distress (1 study).<br><br>-Exposure to floods during pregnancy is associated with increased risk of low birth weight (3 studies).<br><br>-Exposure to floods during pregnancy is associated with increased risk of miscarriage (2 studies).<br><br>-Exposure to floods is associated with increased risk of mental health conditions, including PTSD among pregnant women (4 studies).	Risk of bias: no evidence of quality assessment.
Lafortune S (2021).	To perform a meta-analysis to estimate the magnitude of the effects of disaster	- Floods	-Effects of prenatal maternal stress on the offspring (birth outcomes, cognitive development, motor outcomes, physical outcomes, socio-emotional outcomes)	30 studies included in the systematic review and in the meta-analysis, of which 20 focusing on floods.<br><br>-The meta-regression observed a significantly positive overall association between prenatal maternal stress	Systematic review and meta-analysis.<br><br>Risk of bias: Quality assessment

Name of first author and year of publication	Review aim	Disaster type	MNH Outcomes**	Number of included studies & Key Findings	Other Information
	related prenatal maternal stress on different aspects of child development from birth to late adolescence.			and offspring motor outcomes in flood related effect sizes (r = 0.0741; SE= 0.0153; p>0.0001) and a significantly positive overall association between prenatal maternal stress and offspring behavioural outcomes in flood related effect sizes (r = 0.0752; SE= 0.0170; p>0.0001).	conducted for all included studies.
Hwang CH (2021).	To systematically present the findings of the literature on key issues related to infant feeding in emergencies in middle- or high-income countries.	-Hydrometeorological and geophysical disasters	-Effects on infant feeding	30 studies included in the scoping review, of which 15 focusing on hydrometeorological and/or geophysical disasters.<br><br>- The most common challenges for breastfeeding mothers in the context of disasters include lack of privacy or of adequate environment to breastfeed (11 studies), stress/exhaustion (12 studies), limited fluid/nutritious intake for mothers (8 studies), lack of time (4 studies). Additionally challenges include lack of breastmilk expression equipment (1 study) and no electricity for breastmilk storage (1 study).	Scoping review.<br><br>Risk of bias: no evidence of quality assessment.
Rodriguez-Soto (2021).	To systematically present the findings on the association between prenatal maternal stress during pregnancy and childhood temperament through a narrative review approach.	-Hurricanes<br>-Floods<br>-Windstorms	-Child temperament	20 studies included in the systematic review, of which 3 focused on windstorms, 2 on floods and 1 on hurricane.<br><br>-Exposure to windstorms during pregnancy is associated with increased risk of negative affectivity in the offspring (3/3 studies.<br><br>-Exposure to floods during pregnancy is associated with increased risk of greater toddler reactivity (2 studies).<br><br>-Exposure to hurricanes and flooding during pregnancy is associated with increased risk of difficult temperament at 12 months (1 study).	Risk of bias: Quality assessment conducted for all included studies.

MNH: maternal and newborn health. PTB: preterm birth. LBW: low birth weight. PTSD: post-traumatic stress disorder. SGA: small for gestational age.

* Timing of exposure varies; method of measurement/assessment of the exposure varies.

**The provided definitions of MNH outcomes are aligned with established definitions in the literature, unless otherwise specified.

Table S4d. Reviews on water security, quality and availability and MNH (N=1).

Name of first author and year of publication	Review aim	Climate related exposure*	MNH Outcomes**	Number of included studies & Key Findings	Other Information
Talukder MRR (2017).	To systematically review epidemiological studies that examined sodium content in water and blood pressure levels and to perform a meta-analysis.	-Water salinity (drinking water)	-Hypertensive disorders of pregnancy= 3 studies	10 studies included in the systematic review. 3 studies focused on a pregnant population: the findings of these three studies are reported descriptively given the small number.<br><br>-Inconsistent evidence with regards to the association between drinking water salinity and development of hypertensive disorders of pregnancy.	Systematic review and meta-analysis.<br><br>Risk of bias: Quality assessment conducted for all included studies.

MNH: maternal and newborn health. * Timing of exposure varies; method of measurement/assessment of the exposure varies.

**The provided definitions of MNH outcomes are aligned with established definitions in the literature, unless otherwise specified.

Table S4e. Reviews on food insecurity, nutrition patterns and MNH (N=1).

Name of first author and year of publication	Review aim	Climate related exposure*	MNH Outcomes**	Number of included studies & Key Findings	Other Information
Trudell JP (2021).	To systematically present the findings on the association between food insecurity and mental health in the African continent through a narrative review approach.	-Food insecurity	-Depression during pregnancy or postpartum= 16 studies<br>-Anxiety during pregnancy or postpartum= 4 studies<br>-Mental distress during pregnancy or postpartum= 2 studies	64 studies included in the systematic review, of which 19 focused on pregnant and/or postpartum women.<br><br>-Food insecurity exposure during pregnancy is associated with greater risk of depression (16/16 studies)<br><br>-Food insecurity exposure during pregnancy is associated with greater risk of anxiety (3/4 studies)	Risk of bias: Quality assessment conducted for all included studies.

MNH: maternal and newborn health.

* Timing of exposure varies; method of measurement/assessment of the exposure varies.

**The provided definitions of MNH outcomes are aligned with established definitions in the literature, unless otherwise specified.

Online Supplementary Document S5

Table S5. Heat exposure/ seasonality and MNH outcomes: summary of reported associations across the included systematic reviews.

Summary of reported associations between heat exposure and/or hot season and MNH outcomes (N=24)				
Heat exposure and *maternal health* outcomes (N=8)*	N of reviews *with evidence* of association	References	N of reviews *without evidence* of association	References
-Hypertensive disorders	3	(19,27,33)	1	(20)
-Gestational diabetes	3	(27,28,37)	0	N/A
-Mental health conditions	0	N/A	1	(39)
-Access to maternal health services	1	42	0	N/A
Heat exposure and *fetal/perinatal health* outcomes (N=14)	N of reviews *with evidence* of association	References	N of reviews *without evidence* of association	References
-Preterm birth	12	(19-23,26,27,31,32, 34,35,39)	0	N/A
-Stillbirth	8	(21-24, 26,27,32)	0	N/A
-Congenital anomalies	2	(25,26)	2	(20,27)

-Miscarriage	0	N/A	1	(27)
Heat exposure and *newborn health* outcomes (N=15)	N of reviews *with evidence* of association	References	N of reviews *without evidence* of association	References
-Birth weight variations	10	(19,21-23, 26, 27, 31, 22,35)	1	(20)
-Infant morbidity	5	(22,27,36,40,41)	0	N/A
-Infant mortality	2	(38,40)	2	(27,31)
-Feeding practices	1	(29)	0	N/A

**The systematic reviews by Ravanelli et al. (2018) and by Megaw et al. (2017) explore respectively limits for heat exposure during pregnancy and ultraviolet exposure. The table reports on common health outcomes, for this reason the aforementioned reviews are not included.*

Online Supplementary Document S6

Table S6. Air pollution exposure and MNH outcomes: summary of reported associations across the included systematic reviews.

Summary of associations between air pollution exposure and MNH outcomes (N=47)				
AAP exposure and *maternal health* outcomes (N=7)	N of reviews *with evidence* of association	References	N of reviews *without evidence* of association	References
-Hypertensive disorders	4	(45,47,60,64)	0	N/A
-Gestational diabetes	2	(44,47)	2	(73,88)
-Mental health conditions	1	(88*)	0	N/A
-Access to health services	1	(88*)	0	N/A
AAP exposure and *fetal/perinatal health* outcomes (N=32)	N of reviews *with evidence* of association	References	N of reviews *without evidence* of association	References
-Preterm birth	15	(35,43,50,51*,53**, 56-58,70-72, 79, 84, 85,87)	4	(48,52,54*,88*)
-Congenital anomalies	7	(56,62,66,75,78,81, 83)	1	(88*)
-Stillbirth	4	(35,44,71,74)	1	(77)
-Miscarriage	4	(59,61,71,80)	0	N/A
AAP exposure and *newborn health* outcomes (N=28)	N of reviews *with evidence* of association	References	N of reviews *without evidence* of association	References

-Birth weight variations	17	(35,52-54, 56-58, 64, 67, 68, 70-72, 76, 79, 84, 87)	4	(43,48,51*,88*)
-Small for gestational age	8	(43,52,56-58, 64, 79,84)	3	(48,54*,72)
-Infant morbidity	7	(55*,56,63,65,69, 82,86)	0	N/A
-Infant mortality	2	(54*,84)	1	(56)
-Feeding practices	1	(88*)	0	N/A

*systematic review investigating wildfire smoke exposure
**systematic review investigating dust storm exposure
